# Integrative analysis of fitness and metabolic effects of a large multidrug-resistant plasmid in Salmonella across diverse serovars

**DOI:** 10.1099/mgen.0.001715

**Published:** 2026-05-18

**Authors:** Yu-Ting Su, Zi-Xuan Li, Peng-Wei Li, Meng-Ting Yang, Ruan-Yang Sun, Ying Xu, Dong Wang, Jian Sun, Xiao-Ping Liao, Liang-Xing Fang

**Affiliations:** 1 National Risk Assessment Laboratory for Antimicrobial Resistance of Animal Original Bacteria, College of Veterinary Medicine, South China Agricultural University, Guangzhou, Guangdong, PR China; 2 Guangdong Provincial Key Laboratory of Veterinary Pharmaceutics Development and Safety Evaluation, South China Agricultural University, Guangzhou, Guangdong, PR China

**Keywords:** fitness costs, multidrug-resistant plasmid, plasmid-chromosome interaction, *Salmonella*

## Abstract

Plasmids, particularly those of the IncHI2 type, are key drivers of multidrug resistance in *Salmonella*. However, the fitness costs imposed by multidrug-resistant (MDR) IncHI2 plasmids across different serovars and the underlying mechanisms remain poorly understood. Here, we report that carriage of the MDR IncHI2 plasmid pMDRHI2 imposes a fitness cost that varies in magnitude across *Salmonella* hosts. This phenotypic variation is accompanied by serovar-specific differences in both transcriptional and metabolic reprogramming. Notably, despite this heterogeneity, plasmid carriage consistently perturbed the expression of chromosomal loci involved in propanediol utilization (*pdu*), ethanolamine utilization (*eut*) and sulphur and glycerophospholipid metabolism, though in a strain-dependent manner. Integrative transcriptomic and metabolomic analyses revealed that the fitness costs are linked to distinct metabolic lesions in a serovar-specific manner. Exogenous metabolite supplementation further confirmed that these lesions – such as dysregulated propanoate metabolism or impaired redox homeostasis – underlie the observed fitness costs. Collectively, these findings demonstrate that the fitness burden imposed by MDR IncHI2 plasmid carriage is dictated by serovar-specific host factors and arises from plasmid-mediated remodelling of central metabolic pathways.

**Impact Statement**

The escalating incidence of multidrug-resistant (MDR) *Salmonella* infections is a critical global public health concern. IncHI2-type plasmids, renowned for their capacity to co-harbour and co-disseminate clinically significant antimicrobial resistance genes, have been instrumental in the proliferation of MDR *Salmonella enterica* – particularly serovars Typhimurium, its monophasic variant 1,4,[5],12:i:-, and Indiana – within food animal and human populations across China and internationally. The fitness costs associated with plasmid carriage are theoretically expected to impede the maintenance of plasmid-bearing bacterial lineages. However, the molecular underpinnings of such costs – especially in the context of large conjugative MDR plasmids like IncHI2 in *Salmonella* – have remained elusive. Here, we found that the MDR IncHI2 plasmid pMDRHI2 imposes a spectrum of fitness costs and elicits divergent transcriptomic and metabolomic profiles across *Salmonella* strains with distinct genetic backgrounds. Crucially, the observed fitness costs are attributable not solely to plasmid-encoded genetic determinants but also to the specific genomic context of the bacterial host, with concomitant alterations in cellular metabolism accompanying these fitness phenotypes. These findings contribute to a more nuanced understanding of plasmid–host interactions and suggest that strategies targeting metabolic reprogramming may offer a viable approach to curbing the dissemination of MDR *Salmonella*.

## Data Availability

All Whole Genome Sequencing (WGS) and transcriptomic data have been deposited in the NCBI database, and their BioProject number is PRJNA1223110. Individual isolate accession numbers and isolate metadata could be found in Table S1 (available in the online Supplementary Material). The research data generated in this study have been deposited in the Figshare repository (https://doi.org/10.6084/m9.figshare.31839619).

## Introduction

Non-typhoidal *Salmonella* (NTS) are serious foodborne pathogens, and the salmonellosis specifically caused by NTS constitutes a major public health concern worldwide. More worryingly, multidrug-resistant (MDR) NTS strains, such as ST19 *Salmonella* Typhimurium, ST34 *Salmonella* 1,4,[5],12:i:- (a monophasic variant of *Salmonella* Typhimurium) and ST17 *Salmonella* Indiana, are continually emerging and have now disseminated on a global scale [1,5]. These MDR *Salmonella* strains exhibit high-level resistance to front-line fluoroquinolones and third-generation cephalosporins and have even developed resistance to colistin and carbapenems – the last therapeutic options for treating MDR *Enterobacteriaceae* infections. Consequently, infections caused by such MDR *Salmonella* strains have become a severe threat to both food animals and humans. Of note, IncHI2 plasmids, which are characterized by their large size (typically >200 kb), are widespread among *Enterobacteriaceae* and are particularly prevalent in *Salmonella* spp. These plasmids frequently co-harbour and co-spread clinically important antibiotic resistance genes (ARGs), driving the rapid emergence of MDR *Salmonella* strains in both food animals and humans across China and other countries [1,3, 5,7].

Despite the potential adaptive benefits they may confer, plasmids often impose a fitness cost on their hosts, particularly in environments lacking selection for plasmid-encoded traits [8]. These costs can arise during different stages of the plasmid’s residence within the host [9]. Generally, the fitness costs associated with plasmid carriage stem from three main factors [9,13]: (i) the metabolic burden of plasmid replication and the expression of plasmid-encoded genes; (ii) the interference of plasmid-encoded genes with specific host genes, leading to deleterious interactions between newly acquired genes and bacterial regulatory networks; and (iii) cytotoxicity resulting from plasmid-encoded gene products. The fitness cost imposed by plasmid carriage is likely a major obstacle to plasmid persistence in bacterial populations. However, the underlying molecular mechanisms by which large conjugative MDR plasmids inflict fitness costs remain poorly understood.

Here, we combined phenomics, transcriptomics and metabolomics to investigate the fitness costs exerted by a 251.056 kb MDR IncHI2 plasmid, pMDRHI2, and its potential underlying molecular mechanisms in a collection of five *Salmonella* strains spanning four serovars. Our results demonstrated that plasmid pMDRHI2 imposed a broad range of fitness costs and induced distinct transcriptomic and metabolomic profiles in *Salmonella* strains with different genetic backgrounds. The fitness costs associated with plasmid carriage stemmed not only from the genetic basis of the MDR plasmid itself, but also from the specific genetic background of the hosts.

## Methods

### Bacterial strains, plasmid and conjugation

All bacterial strains used in this study are listed in Table 1. Plasmid pMDRHI2 is a variant of IncHI2 plasmid pJXP9 from *Salmonella enterica* serovar 1,4,[5],12:i:- JXP9 [14]. The two plasmids differ only in their MDR regions. Like pJXP9, pMDRHI2 possesses a typical IncHI2 backbone and harbours 15 ARGs, including the plasmid-mediated colistin-resistance gene *mcr-1*, the extended-spectrum *β*-lactamase (ESBL) gene *bla*_CTX-M-14_ and the plasmid-mediated quinolone resistance gene *oqxAB*.

**Table 1.** Strains used in this study

Name	Serovar	MLST	Year	Location	Origin
JXP9	1,4,[5],12:i:-	34	2017	Jiangxi	Swine
ZJM302	1,4,[5],12:i:-	34	2015	Guangdong	Chicken
L-S3359	1,4,[5],12:i:-	34	2012	Guangdong	Human
D110	Typhimurium	19	2018	Shandong	Chicken
25FS15	Indiana	17	2020	Guangdong	Duck
45	Enteritidis	11	2020	Henan	Duck

Five *Salmonella* isolates, representing four distinct serovars, were used as recipient strains for conjugation. Spontaneous rifampicin-resistant mutants of these isolates were selected (MIC ≥100 µg ml^−1^) to facilitate counterselection. Conjugal transfer of pMDRHI2 was performed using liquid mating experiments with *S*. 1,4,[5],12:i:- JXP9/pMDRHI2 as the donor strain. We selected the transconjugants on Luria-Bertani (LB) agar plates supplemented with rifampicin (50 µg ml^−1^) and fosfomycin (64 µg ml^−1^).

### Whole-genome sequencing and mutation analysis

Both transconjugants and their corresponding rifampicin-resistant recipients were subjected to whole-genome sequencing using the Illumina HiSeq platform (San Diego, CA, USA). Raw sequence reads were assembled into contigs using SPAdes v3.6.2 with the following parameters: -k 21,33,55,77,99,127 --careful --phred-offset 33 [15]. Assembly quality metrics were assessed using Quast v5.2.0 [16]. To identify mutations acquired during conjugation, we identified SNPs in each transconjugant by mapping sequencing reads to the corresponding plasmid-free recipient genome using Snippy v4.6.0 (https://github.com/tseemann/snippy).

### Growth curves *in vitro*

Overnight cultures of bacterial strains grown in LB medium at 37 °C were diluted in PBS to an OD at 600 nm (OD₆₀₀) of 0.1. Aliquots (1 ml) of each diluted culture were dispensed in quadruplicate into 48-well plates. The plates were then placed in an Ensight multimode plate reader (PerkinElmer, Burlington, MA, USA) and incubated in LB medium at 37 °C with continuous shaking at 180 r.p.m. Bacterial growth was monitored by measuring OD₆₀₀ at regular intervals for a minimum of 24 h. From the resulting growth curves, three parameters were extracted as proxies for bacterial fitness: (i) maximum growth rate, (ii) area under the growth curve (AUC) and (iii) lag phase duration (lag time). The AUC and maximum growth rate were calculated using the *growthcurver* package in R. The lag time was determined using Origin 2021 (OriginLab Corporation, Northampton, MA, USA).

### Competition assays *in vitro*

The competitive fitness of plasmid-carrying transconjugants (hereafter referred to as ‘transconjugants’) relative to their plasmid-free parental strains (hereafter referred to as ‘plasmid-free counterparts’) was assessed using *in vitro* indirect competition assays, performed as described previously [17]. In preliminary liquid mating experiments, we observed that pMDRHI2 transferred from the donor strain *S*. 1,4,[5],12:i:- JXP9/pMDRHI2 to *S*. Kentucky strain a55 at an extremely low frequency (~10^−^¹¹; data not shown), indicating that a55 is a poor recipient for this plasmid. To minimize the risk of plasmid transfer during co-cultivation, we therefore employed *S*. Kentucky a55 as a common competitor strain in all competition assays, co-culturing it separately with each pMDRHI2-carrying transconjugant and its corresponding plasmid-free recipient.

To enable differential enumeration of the competing strains, fluorescent reporter systems were introduced. The *mCherry* (red fluorescent protein) gene was cloned into the pUC19 vector and introduced into all pMDRHI2-carrying transconjugants and their plasmid-free counterparts via electrotransformation. The *gfp* (green fluorescent protein) gene was similarly cloned into pUC19 and introduced into the competitor strain a55. This dual-fluorescence labelling allowed precise quantification of each population during co-culture.

The relative fitness (RF) of transconjugants was assessed using direct *in vitro* competition assays performed in triplicate, following previously described protocols with slight modifications [18]. Two parallel competitions were conducted: (i) transconjugants (pMDRHI2-carrying, pUC19-*mCherry*-labelled) versus the common competitor strain a55 (pUC19-*gfp*-labelled) and (ii) the corresponding plasmid-free recipients (pUC19-*mCherry*-labelled) versus the same a55-pUC19-*gfp* competitor. For each competition, overnight cultures grown in LB medium at 37 °C were diluted to an OD₆₀₀ of 0.1. The two competing strains were then mixed in a 1 : 1 ratio in 5 ml of fresh LB broth within 50 ml centrifuge tubes and incubated at 37 °C for 24 h. At both the initial (0 h) and final (24 h) time points, aliquots were collected, serially diluted and analysed using an Attune NxT flow cytometer (Thermo Fisher, Pittsburgh, PA, USA). Bacterial populations were distinguished based on size gating and fluorescent signal thresholds. Red fluorescent cells (pUC19-*mCherry*-labelled) were excited using a 561 nm laser, while green fluorescent cells (pUC19-*gfp*-labelled) were excited using a 488 nm laser. c.f.u. were determined for each population at both time points.

RF was calculated using the following formula: RF₁ (for transconjugants vs. a55) or RF₂ (for recipients vs. a55)=(log₁₀S1_dt−log₁₀S1_d0)/(log₁₀S2_dt−log₁₀S2_d0). S1 and S2 represent the c.f.u. densities of the two competing strains, and d0 and dt denote the initial and final time points (in days), respectively. The RF of transconjugants compared to their plasmid-free counterparts was then inferred from the ratio RF₁/RF₂. An RF value greater than one indicates a selective advantage of the test strain over the competitor, whereas an RF value less than one signifies a fitness cost.

### Transcriptome profiling and analysis

Transcriptome profiles were generated for selected transconjugants and their plasmid-free counterparts. Bacteria were cultured in LB broth and harvested at the exponential growth phase as previously described [19]. For each strain, three independent biological replicates were prepared. Total RNA was extracted, and barcoded RNA libraries were constructed via reverse transcription. Sequencing was performed on the Illumina HiSeq 2000 platform at Majorbio (Shanghai, China). After quality trimming, the resulting reads were aligned to the corresponding recipient *Salmonella* genome using Bowtie2 with default parameters. Gene expression levels were quantified using the FPKM (Fragments Per Kilobase of transcript per Million mapped reads) method. Differentially expressed genes (DEGs) were identified based on the following criteria: adjusted *P*-value <0.05 and |log₂ fold change (FC)|>1. Functional annotation and enrichment analysis of chromosomal DEGs were performed using Gene Ontology (GO; http://geneontology.org/) and KEGG pathway databases (http://bioinfo.org/kobas/).

### Quantitative reverse transcription PCR validation

To validate the transcriptomic data, quantitative reverse transcription PCR (RT-qPCR) was performed on selected genes. Bacterial cultures were grown to the exponential phase, and total RNA was extracted from cell pellets using the Bacterial RNA Extraction Kit (Omega). Reverse transcription was carried out with the HiScript III 1st Strand cDNA Synthesis Kit (+gDNA Wiper) (Vazyme, Nanjing, China) to generate cDNA templates. We performed RT-qPCR using the Taq Pro Universal SYBR qPCR Master Mix (Vazyme) with gene-specific primers (listed in Table S2). The thermal cycling conditions are detailed in Table S3. All reactions were conducted in triplicate for each biological sample. The 16S rRNA gene was used as an internal reference to normalize gene expression levels. Relative expression levels were calculated using the 2^–ΔΔCt^ method.

### Metabolomics

Untargeted metabolomic analysis was performed on transconjugants and their plasmid-free counterparts using ultra-HPLC-MS/MS, as previously described [20]. Six biological replicates were analysed for each group to capture metabolic changes associated with plasmid carriage. Sample preparation and metabolite extraction were conducted at Novogene (Beijing, China). Briefly, samples were precipitated and analysed using a Vanquish UHPLC system (Thermo Fisher, Germany) coupled with an Orbitrap Q Exactive HF mass spectrometer (Thermo Fisher, Germany). Detection was performed in both positive and negative ion modes to maximize metabolite coverage.

We processed raw data files using Compound Discoverer v3.3 (Thermo Fisher) for peak alignment, peak picking and metabolite quantification. Key processing parameters included peak area correction based on the first quality control sample, mass tolerance of 5 ppm, signal intensity tolerance of 30% and minimum intensity threshold filtering. Normalized data were used to predict molecular formulas based on additive ions, molecular ion peaks and fragment ions. Metabolites were identified by matching mass spectra against the mzCloud (https://www.mzcloud.org/), mzVault and MassList databases, yielding both qualitative and relative quantitative information. Annotations were further enriched using the KEGG, HMDB and LIPID Maps databases.

We performed multivariate statistical analyses, including principal component analysis and partial least squares discriminant analysis, using the metaX package [21]. Univariate analysis (t-test) was applied to assess statistical significance. Metabolites meeting the following criteria were considered differentially expressed metabolites (DEMs): variable importance in projection (VIP) >1, *P*-value<0.05, and |FC|≥1.2 or ≤0.833. Functional enrichment and pathway analyses of DEMs were conducted using the KEGG database.

### Integration and correlation analysis of transcriptomic and metabolomic data

To investigate the coordinated changes and potential associations between metabolites and genes in response to plasmid carriage, we performed an integrative correlation analysis. For each strain pair (transconjugant versus their plasmid-free counterparts), the 40 most significantly altered DEMs (VIP >1, |FC|≥1.2 or ≤0.833, *P*<0.05) and the major DEGs (|FC|≥2 or ≤0.5, *P*<0.05) were selected for network analysis. Pearson correlation coefficients (r) between DEMs and DEGs were calculated using the *cor* function from the *psych* package in R (https://cran.r-project.org/package=psych). Only correlations with |r|>0.80 and *P*<0.05 were retained for network construction. The resulting correlation networks were visualized using the Metware Cloud platform (https://cloud.metware.cn).

### Addition of DEMs

To test whether altered metabolite levels contribute to the fitness costs of pMDRHI2 carriage, we supplemented plasmid-free recipients with exogenous DEMs at specified concentrations and measured growth kinetics as described above.

## Results

### Growth and competition fitness costs of plasmid carriage

The IncHI2 plasmid pMDRHI2 was successfully transferred via conjugation into five rifampicin-resistant *Salmonella* recipients, representing four distinct serovars. WGS confirmed the presence of the *repHI2* gene in all resulting transconjugants. In addition, comparative genomic analysis revealed mutations within coding regions in each of the five transconjugants relative to their corresponding plasmid-free parental strains. Four transconjugants carried one to three mutations – including non-synonymous substitutions, frameshifts, stop-gain and initiator codon alterations – within functional genes or those encoding hypothetical proteins. The remaining transconjugant (ZJM302/pMDRHI2) exhibited a higher mutation burden, with non-synonymous or frameshift mutations identified in 21 genes, comprising 16 functional genes and 5 genes encoding hypothetical proteins (Data S1).

**Fig. 1.**
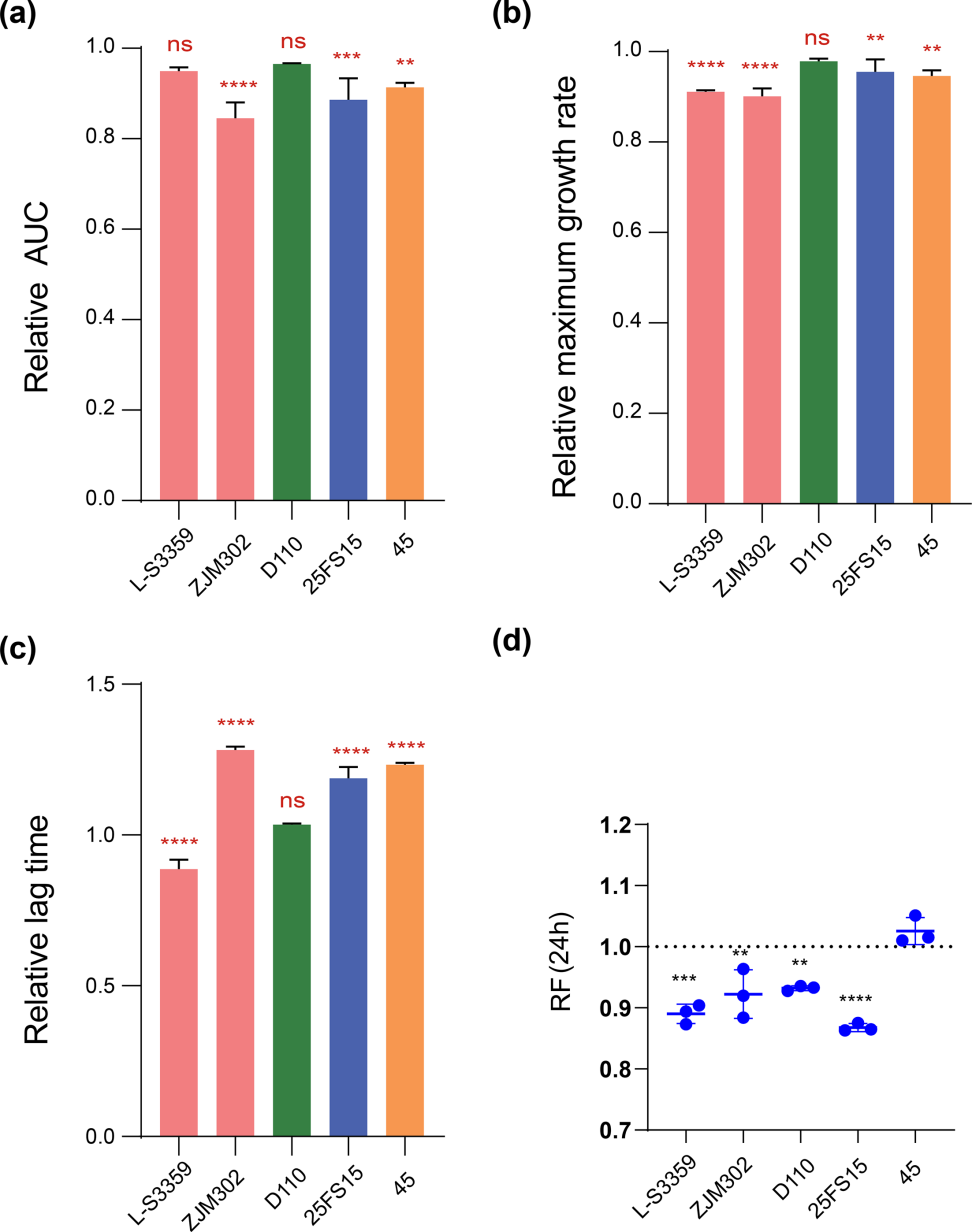
The growth and competition fitness costs associated with pMDRHI2 plasmid carriage in *Salmonella* across different serovars. (**a**) Relative AUC, (**b**) growth rate and (**c**) lag time of plasmid-carrying *Salmonella* strains against the corresponding plasmid-free *Salmonella* strains; (**d**) relative competition fitness of plasmid-carrying *Salmonella* strains against the corresponding plasmid-free *Salmonella* strains.

All five transconjugants displayed visible growth disadvantages in the growth curve profiles compared to their plasmid-free counterparts (Fig. S1). To quantitatively assess growth costs, three parameters were analysed: area under the curve (AUC), maximum growth rate and lag time. The AUC was significantly reduced (*P*<0.05) for three transconjugants relative to their plasmid-free equivalents, whereas no significant difference (*P*>0.05) was observed for the remaining two strains (Fig. 1a). The relative maximum growth rate was also significantly reduced (*P*<0.05) in four of the five transconjugants (Fig. 1b). Analysis of lag phase duration showed a significant extension in three transconjugants (ZJM302/pMDRHI2, 25FS15/pMDRHI2 and 45/pMDRHI2) relative to their plasmid-free parents, while the transconjugant L-S3359/pMDRHI2 displayed a significantly shorter lag time than its plasmid-free recipient (Fig. 1c). For strain D110 harbouring pMDRHI2, no significant difference (*P*>0.05) was observed in either lag time or maximum growth rate. Collectively, these quantitative assessments revealed that growth costs were evident in four of the five transconjugants, with strain D110 being the only one that did not show significant deviations in any of the three parameters analysed.

Indirect competition assays conducted over 24 h demonstrated that the plasmid-free parental strain achieved higher cell yields than the corresponding transconjugant in four of the five strain pairings examined (Fig. 1d). Notably, a substantial fitness cost was observed for plasmid carriage in strains 25FS15 and L-S3359, whereas a comparatively modest cost was evident in strains ZJM302 and D110. In contrast, plasmid pMDRHI2 imposed no measurable competitive fitness burden in strain 45. In summary, carriage of pMDRHI2 imposed either a growth or competitive fitness cost in all five *Salmonella* recipients examined, with particularly pronounced costs observed in strains ZJM302 and 25FS15.

### Transcriptome profile changes in *Salmonella* associated with plasmid carriage

**Fig. 2.**
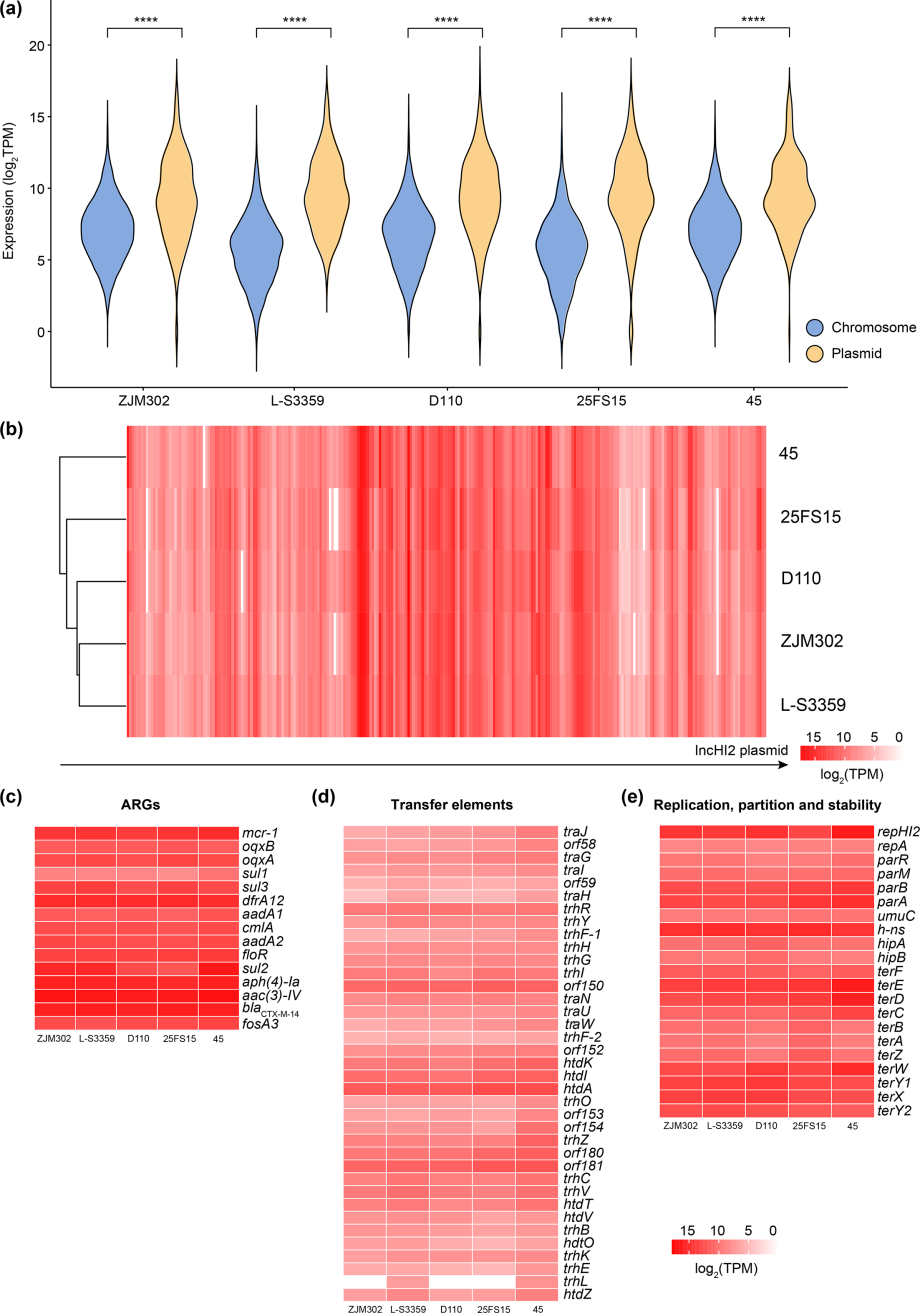
Characteristics of chromosome-encoded and plasmid-encoded gene expression in *Salmonella* across different serovars. (**a**) Transcript abundance in log_2_ of transcripts per million (TPM) of chromosome-encoded (blue) and plasmid-encoded (orange) genes in each of pMDRHI2 plasmid-carrying *Salmonella* strains; (**b**) cluster analysis of plasmid transcriptome profiles through transcript abundance (TPM) of plasmid-encoded genes in each of pMDRHI2 plasmid-carrying *Salmonella* strains; (**c–e**) transcript abundance (TPM) of pMDRHI2 plasmid-encoded (**c**) ARGs, (**d**) transfer elements and (**e**) replication, partition and stability genes.

To investigate the origin of the fitness costs associated with plasmid pMDRHI2 in *Salmonella* hosts of differing serovars, we performed transcriptomic profiling via RNA-Seq on each of five strain pairs. In all cases, plasmid-encoded genes were expressed at significantly higher levels than chromosomal genes (*P*<0.0005; Fig. 2a; Data S2 and S3), consistent with observations reported previously [11]. Notably, 17 chromosomal genes exhibited very high expression levels [log₂ TPM (transcripts per million) >14] in at least 1 of the 5 transconjugants. These included genes involved in heat/cold shock and stress responses (*cspC*, *ibpB*, *ibpA*, *grcA* and *bhsA*), ribosome function and translation (*rpmI*, *rplT*, *infC* and *raiA*) and outer membrane proteins (*lpp*, *lppB*, *ompA* and *ompD*), as well as *ahpC_1* (alkyl hydroperoxide reductase C) and 5 genes encoding hypothetical proteins (Table S4).

Expression of plasmid-encoded genes was largely strain-dependent, though similar profiles were observed between *S*. 1,4,[5],12:i:- strains ZJM302/pMDRHI2 and L-S3359/pMDRHI2 (Fig. 2b). Across nearly all five transconjugants, ARGs and genes involved in plasmid replication, partitioning and stability were among the most highly expressed, whereas transfer-associated elements were expressed at relatively low levels (Fig. 2c–e). The 12 most highly expressed plasmid genes (log_2_TPM>14) included ARGs [*aac(3)-IV*, *bla*_CTX-M-14_, *aph(4)-Ia*, *dfrA12*, *mcr-1 *and *sul2*], plasmid replication and stability genes (*repHI2*, *hn-s*) and other genes (*nimC*, *estX*, IS*6* and *orf82*). With the exception of *sul2*, IS*6* and *repHI2*, nearly all of these highly expressed plasmid genes were consistently expressed at high levels across all five *Salmonella* strains (Table S5).

We next compared plasmid-derived gene expression among *Salmonella* hosts of differing serovars, using the transconjugant D110/pMDRHI2 (*S*. Typhimurium) as a reference, given the relatively low fitness cost associated with plasmid carriage in this strain. Several plasmid-encoded genes exhibited significantly differential expression in the other four strains (*S*. 1,4,[5],12:i:- ZJM302 and L-S3359, *S*. Indiana 25FS15 and *S*. Enteritidis 45) relative to D110/pMDRHI2. The number of DEG numbers ranged from 19 to 56 across the four comparisons (Fig. S2A–D, Data S4). These DEGs included genes involved in plasmid replication, transfer, partitioning and stability, as well as ARGs, mobile genetic elements and genes encoding hypothetical proteins (Fig. S2E–G). DEGs related to plasmid transfer and tellurium resistance were differentially expressed in all four comparisons, and the plasmid partitioning gene *parA* and toxin–antitoxin system *hipA/B* were differentially expressed in two and three comparisons, respectively (Fig. S2F). The *repHI2* gene was significantly up-regulated in 45/pMDRHI2 relative to D110/pMDRHI2 but significantly downregulated in 25FS15/pMDRHI2 relative to D110/pMDRHI2. The sulphonamide resistance gene *sul2* was significantly upregulated in three of the four comparisons, with the exception being 25FS15/pMDRHI2 versus D110/pMDRHI2, where no plasmid-encoded ARGs showed differential expression (Fig. S2E). Additionally, the streptomycin 3′-adenylyltransferase gene *aadA* and the sulphonamide resistance gene *sul3* were significantly upregulated in ZJM302/pMDRHI2 versus D110/pMDRHI2 and 25FS15/pMDRHI2 versus D110/pMDRHI2, respectively.

**Fig. 3.**
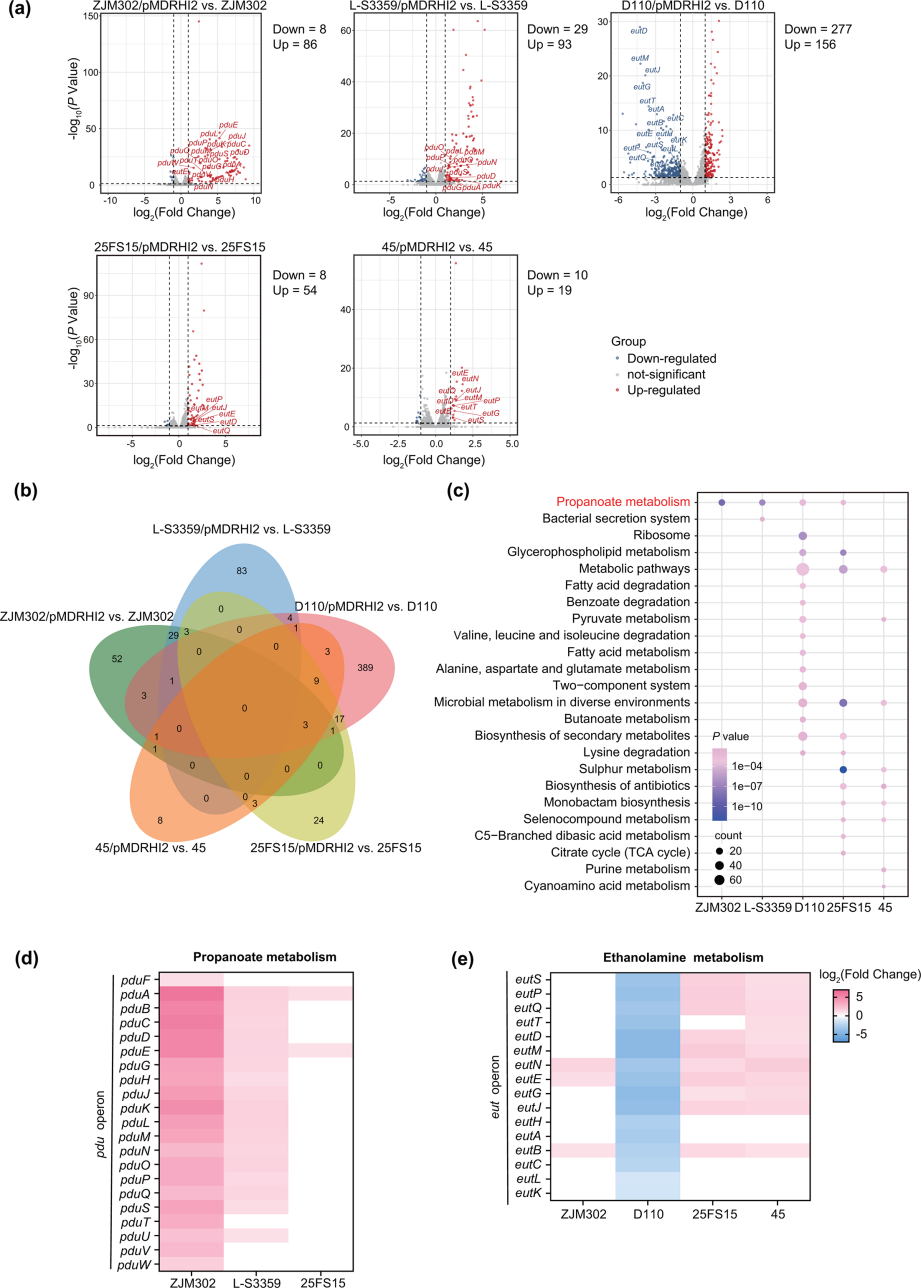
Change in chromosomal gene expression in response to plasmid carriage. (**a**) Chromosomal transcriptome profiles for pMDRHI2 plasmid-carrying *Salmonella* strains relative to that of the corresponding plasmid-free *Salmonella* strains. (**b**) Shared numbers of DEGs between the indicated strains. (**c**) KEGG enrichment analysis using all DEGs from five pMDRHI2 plasmid-carrying *Salmonella* strains relative to that of the corresponding plasmid-free *Salmonella* strains; the specific pathway related to propanediol utilization was marked in red. (**d, e**) Gene expression analysis of (**d**) the propanediol utilization operon (*pdu*) and (**e**) the ethanolamine utilization operon (*eut*).

We further examined the impact of pMDRHI2 carriage on chromosomal gene expression in each of five strain pairs. The presence of pMDRHI2 significantly altered the expression of numerous chromosomal genes in all five strains (Fig. 3a; Data S5). The largest number of DEGs was observed in *S*. Typhimurium D110 (*n*=433), while DEG numbers ranged from 29 to 122 in the remaining four strains. Importantly, no DEGs were identified as common to all five test strains, highlighting the strain-specific nature of the transcriptional response (Fig. 3b). To identify broader transcriptional shifts, we performed gene set enrichment analyses. GO annotation analysis revealed that DEGs in *S*. 1,4,[5],12:i:- strains ZJM302 and L-S3359 were associated with biological processes such as propanediol and diol catabolism, cellular components, including bacterial microcompartments and propanediol degradation polyhedral organelles, and molecular functions related to oxidoreductase activity. In contrast, DEGs in the remaining three strains (D110, 25FS15 and 45) were enriched for ethanolamine catabolic processes and associated bacterial microcompartments (Fig. S3). KEGG pathway enrichment analysis indicated distinct pathway profiles for each strain, with propanoate metabolism being the most commonly affected pathway (four of five strains; Fig. 3c). Specifically, pMDRHI2 carriage significantly upregulated the propanediol utilization (*pdu*) operon in *S*. 1,4,[5],12:i:- strains ZJM302 and L-S3359 (Fig. 3d). In contrast, the ethanolamine utilization (*eut*) operon was significantly upregulated in *S*. Enteritidis strain 45 and *S*. Indiana strain 25FS15 but downregulated in *S*. Typhimurium D110 (Fig. 3e). The reliability of the transcriptomic data was validated by qRT-PCR for selected *pdu* and *eut* genes in strains ZJM302 and D110, respectively (Fig. S4). To exclude the possibility that variations in expression were due to pseudogenization or loss of function, we compared the *pdu* and *eut* operons across the five strains with those of the reference *S*. Typhimurium ATCC 14028. Sequence analysis revealed high nucleotide identity (97.87%–99.98%) and coverage (91.6%–100%) across all strains (Table S6; Fig. S5). Notably, the *eut* operons in *S*. Enteritidis 45 and *S*. Indiana 25FS15 showed slightly lower identity (~98%) and coverage (~91%) relative to ATCC 14028.

Additional transcriptional changes included significant upregulation of sulphur metabolism genes in strains 25FS15 (*cysJ*/*I*/*H*/*D*/*N*/*K*/*W*/*U*/*P*) and 45 (*cysI*/*D*/*N*), as well as upregulation of glycerophospholipid metabolism genes (*glpA*/*B*/*C*/*T*/*Q*) in strains 25FS15 and D110. In strain D110, genes involved in l-carnitine metabolism (*caiTABCDE*, *fixABCX*), hydrogenase 3 components (*hycB*/*C*/*D*/*E*/*G*/*H*/*I*), periplasmic nitrate reductase (*napADFGH*) and multiple ribosomal proteins (*rpsQ, rpmC*, *rplP*, *rpsC*, *rplV*, *rpsS*, *rplB*/*W*/*D*/*C*/*J* and *rpsJ*) were significantly downregulated. Conversely, the l-lactate utilization operon (*lldPRD*) was significantly upregulated in this strain (Data S5).

In summary, our findings reveal both serovar- and host-specific effects on chromosomal and plasmid gene expression in pMDRHI2-carrying *Salmonella* strains. Despite this variability, a common set of chromosomal genes – including those involved in heat/cold shock and stress responses, ribosome and translation functions and outer membrane components – were consistently expressed at higher levels than that of other chromosomal genes with each strain, and a pattern observed across all serovars. Similarly, plasmid-encoded ARGs, *repHI2* and *h-ns* were expressed at higher levels than plasmid transfer elements. More importantly, the transcriptional response of the bacterial host to pMDRHI2 carriage was also strain- and serovar-dependent. The alterations in the expression of chromosomal genes involved in propanoate and ethanolamine metabolism were associated with pMDRHI2 carriage and appeared to be largely independent of host genetic background. Finally, compared with the reference strain D110, a subset of plasmid-encoded genes – including ARGs and those involved in plasmid transfer and stability – were differentially expressed in the other four strains, highlighting the influence of host background on plasmid gene regulation.

### Metabolism profile changes in *Salmonella* associated with plasmid carriage

**Fig. 4.**
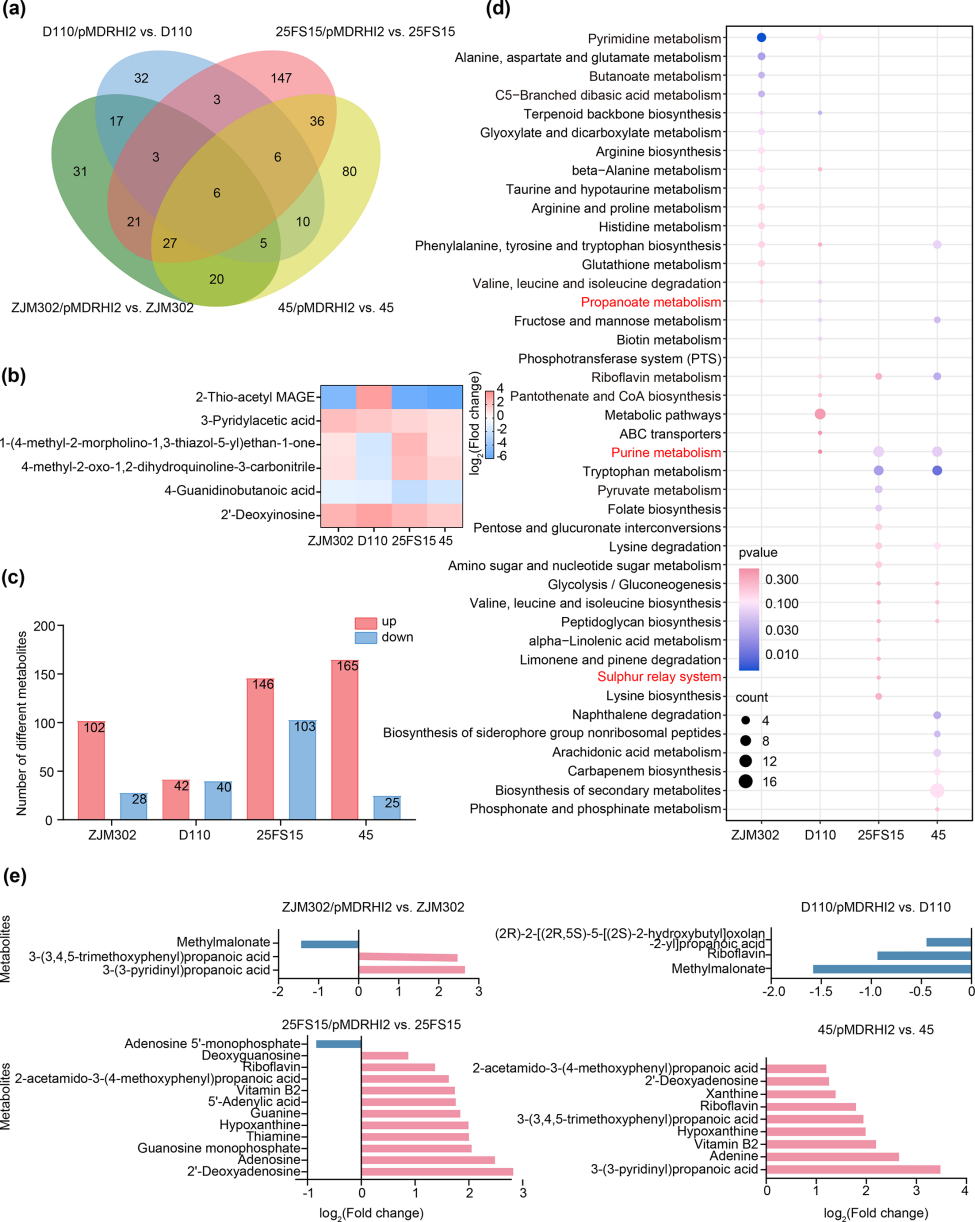
Alterations in metabolomic profiles in response to plasmid carriage. (**a**) Shared numbers of differential metabolites between the indicated strains. (**b**) The abundance of six differential metabolites shared in each of pMDRHI2 plasmid-carrying *Salmonella* strains relative to that of the corresponding plasmid-free *Salmonella* strains. (**c**) The number of differential metabolites from each of pMDRHI2 plasmid-carrying *Salmonella* strains relative to that of the corresponding plasmid-free strains; ‘up’ means differential metabolites increased abundance, and ‘down’ means differential metabolites decreased abundance. (**d**) KEGG enrichment analysis using all differential metabolites from each of pMDRHI2 plasmid-carrying *Salmonella* strains relative to that of corresponding plasmid-free *Salmonella* strains; the specific pathways were marked in red (**e**) showing the abundances of differentially metabolites that were enriched to specific metabolic pathways.

To investigate how pMDRHI2-mediated alterations in the expression of metabolic genes – particularly those involved in propanoate and ethanolamine metabolism – impact the metabolic profiles of plasmid-carrying clones, we performed HPLC-MS-based metabolomic analysis on four pMDRHI2-carrying *Salmonella* strains representing distinct serovars: *S*. 1,4,[5],12:i:- strain ZJM302, *S*. Typhimurium strain D110, *S*. Indiana strain 25FS15 and *S*. Enteritidis strain 45. A total of 1,128 metabolites were identified (Data S6). To assess the metabolic impact of plasmid carriage, we compared metabolite abundances in each of four strain pairs. The presence of pMDRHI2 resulted in significant alterations in the abundance of 130, 82, 249 and 190 metabolites in strains ZJM302, D110, 25FS15 and 45, respectively (Fig. 4a, c; Data S7). Notably, a common set of six compounds was consistently altered across all four plasmid-carrying strains. Among these, 3-pyridylacetic acid and 2′-deoxyinosine were consistently increased in abundance in all cases (Fig. 4a, b).

KEGG enrichment analysis revealed that each *Salmonella* host exhibited a distinct pathway-level response to pMDRHI2 carriage. However, in three of the four strains, differentially abundant metabolites (DEMs) were significantly enriched in pathways related to phenylalanine, tyrosine and tryptophan biosynthesis; purine metabolism; and riboflavin metabolism (Fig. 4d). For instance, both riboflavin and vitamin B2 were significantly increased in strains 25FS15 and 45 but significantly decreased in strain D110 (Fig. 4e). Furthermore, several metabolic pathways – including propanoate and purine metabolisms and sulphur relay system – were both found to be enriched in both the metabolome and transcriptome in specific strain backgrounds (Figs3c  4d, e). For example, methylmalonate, a metabolite associated with propanoate metabolism, was significantly reduced in abundance in strains ZJM302 and D110. In contrast, multiple propionic acid derivatives – including 3-(3-pyridinyl)propanoic acid, 3-(3,4,5-trimethoxyphenyl)propanoic acid and/or 2-acetamido-3-(4-methoxyphenyl)propanoic acid – were significantly increased in strains ZJM302, 25FS15 and 45. However, (2R)-2-[(2R,5S)-5-[(2S)-2-hydroxybutyloxolan-2-yl] propanoic acid showed significantly reduced abundance in strain D110 (Fig. 4e). Within purine metabolism, seven of eight DEMs – including 2′-deoxyadenosine, guanosine monophosphate, 5′-adenylic acid, guanine, hypoxanthine, adenosine and deoxyguanosine – were significantly increased across relevant strains, while adenosine 5′-monophosphate was significantly decreased. In strain 45, purine-related metabolites – xanthine, 2′-deoxyadenosine, hypoxanthine and adenine – were also significantly increased. Finally, thiamine, a metabolite linked to the sulphur relay system, was significantly increased in abundance in strain 25FS15.

In summary, our findings reveal host-specific effects on the metabolic profiles of pMDRHI2-carrying *Salmonella* strains. Despite this variability, plasmid carriage consistently altered the abundance of a common subset of metabolites across hosts. Notably, certain DEMs were shared among specific strains, including methylmalonate (associated with propanoate metabolism), riboflavin and vitamin B2 (linked to riboflavin metabolism) and hypoxanthine (associated with purine metabolism). Moreover, several pathways – including propanoate metabolism, purine metabolism and the sulphur relay system – were enriched in both metabolomic and transcriptomic datasets in a strain-dependent manner, suggesting coordinated multi-omic responses to plasmid carriage.

### Integrative transcriptomic and metabolomic profiling uncovers a systemic molecular network associated with plasmid-mediated fitness costs

**Fig. 5.**
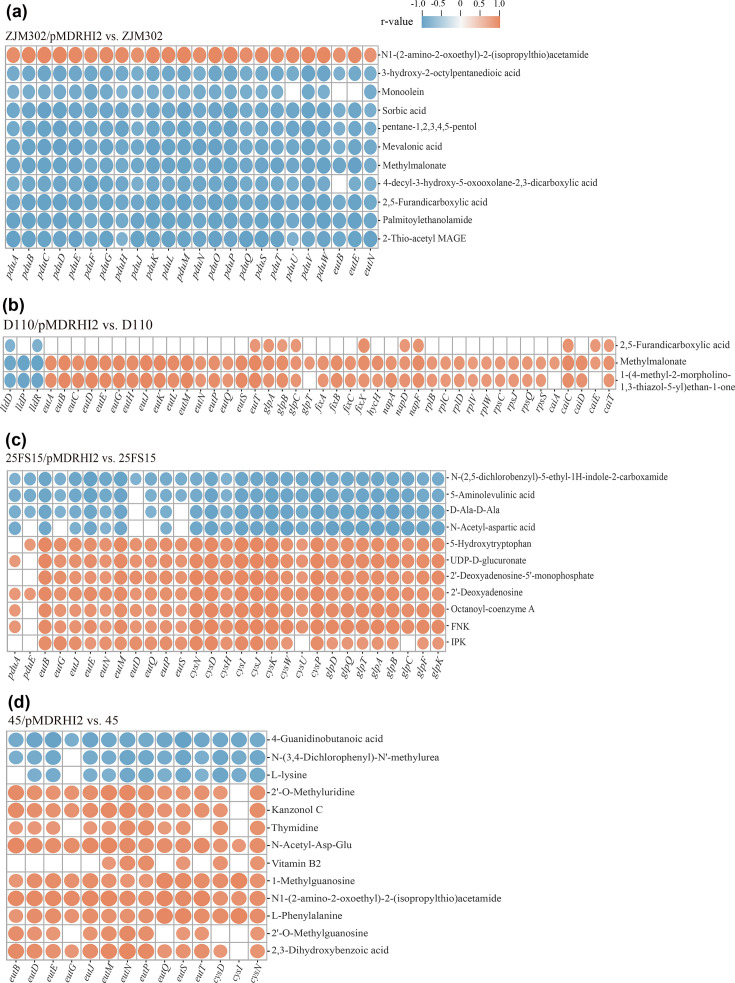
Systematic molecular correlations across multi-omes integrating carriage of plasmid-mediated changes in metabolome and transcriptome. Some of the top 40 DEMs were significantly positively or negatively correlated with most of the major DEGs in each of four pMDRHI2 plasmid-carrying *Salmonella* strains relative to that of corresponding plasmid-free *Salmonella* strains. Statistically significant positive and negative correlations are represented by red and blue circles, respectively.

To explore correlations between metabolites and genes upon plasmid carriage, we integrated transcriptomic and untargeted metabolomic data from corresponding biological samples. For each of the four strain pairs, correlation network analysis (|r|>0.80) was performed on the top 40 significantly altered DEMs and major DEGs (Figs 5, S6 and S7). In the comparison of ZJM302/pMDRHI2 versus ZJM302, the upregulated metabolite N1-(2-amino-2-oxoethyl)-2-(isopropylthio)acetamide exhibited significant positive correlations with all 24 major DEGs, including those of the *pdu* operon. Conversely, 10 downregulated DEMs, including methylmalonate, displayed significant negative correlations with nearly all of these 24 DEGs (Figs 5a, S6A and S7A). In the D110/pMDRHI2 versus D110 comparison, three downregulated DEMs – methylmalonate, 1-(4-methyl-2-morpholino-1,3-thiazol-5-yl)ethan-1-one and 2,5-furandicarboxylic acid – were significantly positively correlated with almost all of the 56 major DEGs (including the *eut*, *cai* and *fix* operons). In contrast, these three DEMs were significantly negatively correlated with genes of the *lld* operon (Figs 5b, S6B and S7B). For the 25FS15/pMDRHI2 versus 25FS15 comparison, seven upregulated DEMs, including 5-hydroxytryptophan, showed significant positive correlations with most of the 29 major DEGs, which included *eut*, *cys* and *glp* genes. However, 4 upregulated DEMs were significantly negatively correlated with the majority of these 29 DEGs (Figs 5c, S6C and S7C). In the 45/pMDRHI2 versus 45 comparison, 10 upregulated DEMs – including vitamin B2 and N1-(2-amino-2-oxoethyl)-2-(isopropylthio)acetamide – exhibited significant positive correlations with nearly all of the 14 major DEGs, which included *eut* and *cys* genes. In contrast, 3 downregulated DEMs showed significant negative correlations with most of these 14 DEGs (Figs 5d, S6D and S7D).

Among the 18 DEMs that were significantly negatively correlated with the majority of major DEGs across the four comparisons, nine were acid compounds with reduced abundance. These included furandicarboxylic acid, 4-decyl-3-hydroxy-5-oxooxolane-2,3-dicarboxylic acid, mevalonic acid, 3-hydroxy-2-octylpentanedioic acid, 2,5-furandicarboxylic acid, 5-aminolevulinic acid, *N*-acetyl-aspartic acid and 4-guanidinobutanoic acid, in addition to methylmalonate (Fig. 5). Among the 17 DEMs that were significantly positively correlated with most major DEGs in 2 comparisons (25FS15/pMDRHI2 vs. 25FS15 and 45/pMDRHI2 vs. 45), 6 were nucleotides or nucleotide derivatives with increased abundance. These included 1-methylguanosine, 2′-deoxyadenosine, 2′-deoxyadenosine-5′-monophosphate, 2′-O-methylguanosine, thymidine and 2′-O-methyluridine (Fig. 5c, d).

Collectively, these correlation analyses suggest that major DEGs may play regulatory roles in key metabolic pathways responding to pMDRHI2 carriage in *Salmonella* hosts of distinct serovars. Notably, across all four comparisons, downregulated acid compounds – particularly methylmalonate – were significantly negatively correlated with most major DEGs, including the *pdu*, *eut* and *lld* operons. In the two comparisons where nucleotide-related changes were prominent (25FS15/pMDRHI2 vs. 25FS15 and 45/pMDRHI2 vs. 45), upregulated nucleotides and their derivatives were significantly positively correlated with most major DEGs, including the *eut*, *cys* and *glp* genes.

### Validation of the metabolic basis of fitness costs via exogenous metabolite supplementation

To investigate whether specific DEMs contributed to the growth fitness costs imposed by pMDRHI2 carriage, we performed exogenous supplementation assays in plasmid-free recipient strains. Candidate metabolites were chosen based on their significant positive or negative correlations with the majority of major DEGs in the corresponding strain backgrounds, as identified through integrated transcriptomic and metabolomic analyses. Strain D110, which did not exhibit a statistically significant growth fitness cost in the quantitative growth parameter analysis, was omitted from this experimental design. Methylmalonate, 5-hydroxytryptophan and vitamin B2 were subsequently validated in strains ZJM302, 25FS15 and 45, respectively.

**Fig. 6.**
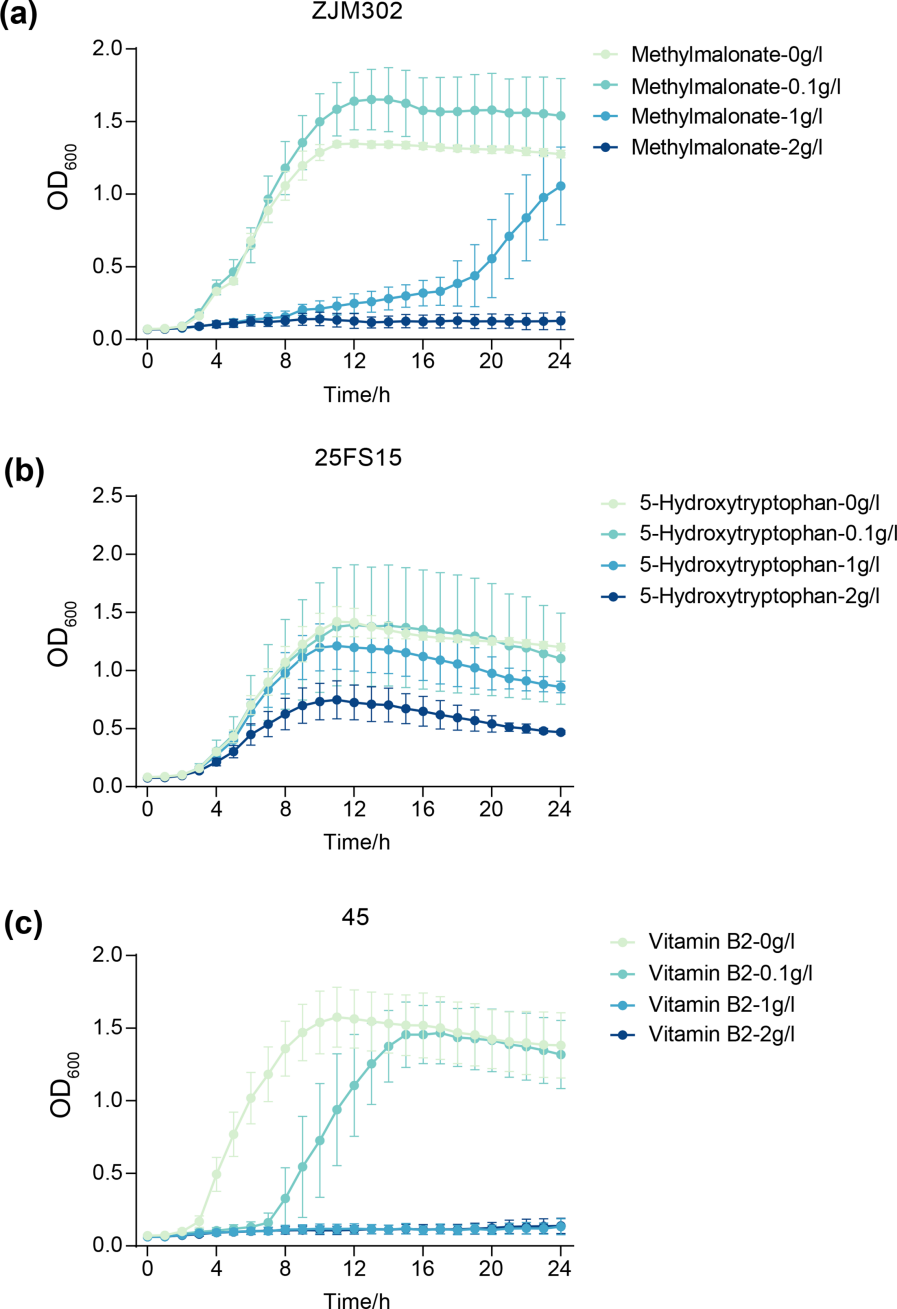
Evaluating the growth fitness costs in plasmid-free *Salmonella* strains with the addition of DEMs.

In strain ZJM302, methylmalonate exhibited a significant negative correlation with most major DEGs. Supplementation with a low concentration of methylmalonate (0.1 g l^−1^) resulted in a modest improvement in growth fitness relative to the unsupplemented control. However, growth was substantially impaired in the presence of intermediate (1 g l^−1^) and high (2 g l^−1^) concentrations of methylmalonate (Fig. 6a). The enhanced growth observed at the lowest concentration is consistent with the reduced endogenous methylmalonate levels observed in the pMDRHI2-carrying ZJM302 transconjugant, suggesting that the plasmid-mediated downregulation of this metabolite may contribute to the fitness cost.

In strain 25FS15, 5-hydroxytryptophan was significantly positively correlated with most major DEGs. Exogenous addition of 5-hydroxytryptophan impaired growth fitness at all three concentrations tested, with inhibitory effects becoming more pronounced as the concentration increased (Fig. 6b). This growth inhibition aligns with the elevated endogenous levels of 5-hydroxytryptophan observed in the pMDRHI2-carrying 25FS15 strain, supporting a causal relationship between metabolite accumulation and reduced fitness.

Similarly, in strain 45, vitamin B2 – which showed significant positive correlations with most major DEGs – was selected for validation. Growth fitness of the plasmid-free strain 45 was reduced upon vitamin B2 supplementation across all tested concentrations, with higher concentrations exerting stronger inhibitory effects (Fig. 6c). This finding is consistent with the increased endogenous vitamin B2 levels detected in the pMDRHI2-carrying 45 transconjugant, further corroborating the role of this metabolite in mediating fitness costs.

Notably, across all three strains, supplementation with intermediate or high concentrations of the respective DEMs uniformly impaired growth fitness, underscoring the concentration-dependent nature of these metabolic effects. Together, these validation experiments provide causal evidence linking specific metabolite perturbations – methylmalonate downregulation in ZJM302, and 5-hydroxytryptophan and vitamin B2 upregulation in 25FS15 and 45, respectively – to the growth fitness costs imposed by pMDRHI2 carriage. These findings further support the integrated multi-omics model in which plasmid-mediated transcriptional reprogramming leads to metabolic imbalances that manifest as measurable growth fitness defects.

## Discussion

Carriage of MDR plasmid pMDRHI2 generally imposed fitness costs across diverse *Salmonella* hosts, with costs varied considerably across serovars and even among strains within the same serovar (Fig. 1). This aligns with previous reports that plasmid properties – such as segregation and conjugation rates, as well as fitness costs – are strongly influenced by host genetic background, with variability observed even in closely related conspecific strains [17,22]. Accordingly, both transcriptional and metabolic responses to pMDRHI2 differed markedly across serovars in our study (Figs 3 and 4). Notably, pMDRHI2 imposed the greatest fitness cost in *S*. 1,4,[5],12:i:- hosts, yet these strains exhibited only moderate alterations in transcriptional and metabolic profiles. Conversely, the smallest fitness cost was in *S*. Enteritidis strain 45, which showed the least transcriptional perturbation but moderate metabolic alteration. These observations suggest that the magnitude of fitness costs does not necessarily correlate with the extent of transcriptional or metabolic remodelling [23,25]. Rather, host genetic background is likely a key determinant of plasmid fitness cost, operating alongside plasmid-specific effects, which was supported by the high genetic heterogeneity among the *Salmonella* recipients.

The fitness costs of MDR plasmids are also thought to arise primarily from the expression of resistance genes themselves [8,26], though the underlying mechanisms remain incompletely understood. Plasmid pMDRHI2 harbours numerous ARGs, including *mcr-1* and *oqxAB*, both of which have been shown to exert deleterious effects on bacterial hosts [27,28]. Consistent with this, several plasmid-encoded ARGs – including *mcr-1* – were expressed at high levels irrespective of host genetic background (Fig. 2c; Table S5). In addition, chromosomal genes encoding outer membrane proteins, heat shock factors and stress response elements were also highly expressed in pMDRHI2-carrying *Salmonella* strains (Table S4). Unexpectedly, these fitness costs – particularly those measured as single-strain growth parameters – were modest (Fig. S1), despite the large size of pMDRHI2 and its carriage of multiple ARGs, including *mcr-1* and *oqxAB*. Interestingly, pMDRHI2 encodes H-NS, a nucleoid-associated protein known to silence transcription of mobile genetic elements, including plasmids [29,30], thereby partially mitigating the detrimental effects of plasmid carriage on bacterial fitness. Additionally, all transconjugants acquired several chromosomal mutations – mostly nonsynonymous substitutions – within functional genes, which may also contribute to the observed fitness costs. A limitation is that with only one transconjugant clone per strain, we cannot separate plasmid effects from conjugation-associated mutations – an issue future studies with multiple independent transconjugants could address. Collectively, our results underscore the complex interplay between plasmid-encoded elements and host genomic context in shaping fitness.

Plasmid carriage significantly altered the expression of a conserved set of metabolic genes, including marked upregulation of the *pdu* or *eut* operon in all plasmid-carrying strains except *S*. Typhimurium D110 (Fig. 3d, e). The *pdu* and *eut* operons are required for the anaerobic utilization of 1,2-propanediol and ethanolamine, respectively – both important carbon sources under oxygen-limited conditions [31,32]. Their upregulation during aerobic growth may reflect an inefficient metabolic adaptation that imposes an energetic burden. Notably, both operons also encode bacterial microcompartment components that sequester toxic intermediates like propionaldehyde and acetaldehyde [33]. Thus, pMDRHI2-induced reprogramming of *pdu*/*eut* operons may compensate for altered energy demands and mitigate cellular disruption caused by toxic ARGs (e.g. *mcr-1* and *oqxAB*). Our findings provide a partial mechanistic explanation for how carriage of the MDR plasmid pMDRHI2 broadly reduces fitness across diverse *Salmonella* strains. Sequence comparison confirmed that the *eut* and *pdu* operons were intact and highly conserved across the tested strains (Table S6; Fig. S5), although non-functional variants cannot be excluded. The absence of functional ethanolamine or propanediol metabolism in a given host might alter fitness consequences and plasmid stability in *Salmonella* populations.

Perhaps the most novel finding of this study is that MDR pMDRHI2 carriage led to overexpression of the *pdu* and *lld* operons in *Salmonella* 1,4,[5],12:i:- and Typhimurium, respectively, altering intracellular propanoate metabolism (Fig. 3). Beyond its canonical role in l-lactate oxidation [34], the *lld* operon also facilitates reduction of l-lactaldehyde to 1,2-propanediol [35], suggesting broader involvement in propanoate-related metabolism in bacterium. Our metabolomic analyses revealed that pMDRHI2 carriage led to reduced levels of methylmalonate and several propionic acid derivatives in both *S*. 1,4,[5],12:i:- and *S*. Typhimurium strains, and these metabolite levels were negatively correlated with *pdu* and *lld* expression (Fig. 4e). Exogenous methylmalonate improved the fitness of plasmid-free *S*. 1,4,[5],12:i:-, indicating that reduced endogenous methylmalonate contributes to fitness costs (Fig. 6). Collectively, these findings link plasmid-induced propanoate metabolism dysregulation to fitness costs in *S*. 1,4,[5],12:i:-.

pMDRHI2 carriage also altered sulphur assimilation, glycerophospholipid metabolism and l-carnitine metabolism in a host-specific manner (Fig. 3c; Data S5). Sulphur metabolic genes (*cysJ*/*I*/*H*/*D*/*N*/*K*/*W*/*T*/*P*) encode cysteine biosynthesis, which was intact and conserved across all tested strains (Table S7). However, non-functional *cys* gene variants have been documented in human-restricted *Salmonella* serovars [36], raising the possibility that an absent sulphur assimilation pathway could alter fitness consequences and plasmid stability in certain hosts. The *glpABC* operon encodes anaerobic glycerol-3-phosphate dehydrogenase. In *E. coli*, electrons from G3P oxidation are transferred to terminal electron acceptors such as fumarate, nitrate or oxygen [37]. Upregulation of this operon in plasmid-carrying strains suggests a shift in respiratory metabolism. The l-carnitine metabolism genes (*caiTABCDE* and *fixABCX* operons) are required by some *Enterobacteriaceae* – including *S*. Typhimurium – to utilize carnitine and crotonobetaine as alternative electron acceptors under anaerobic conditions [38]. Differential expression of these operons further supports that pMDRHI2 carriage altered anaerobic respiration and redox balance in *Salmonella* hosts. Consistent with this, studies on another MDR plasmid (pLL35) have also reported transcriptional and evolutionary changes in host anaerobic metabolism [24,39]. Collectively, these findings indicated that MDR plasmids may actively modulate respiratory metabolism and redox balance to mitigate fitness costs.

Riboflavin is a precursor for Flavin Mononucleotide and Flavin Adenine Dinucleotide, while hypoxanthine may influence redox status. In *S*. Enteritidis and *S*. Indiana, most upregulated DEMs – particularly riboflavin and hypoxanthine – showed strong positive correlations with major DEGs, especially those involved in sulphur and glycerophospholipid metabolism (Figs 5c, d and S7C, D). Exogenous supplementation with the low concentration of riboflavin/hypoxanthine imposed fitness costs on plasmid-free strains (Fig. 6c, d). These findings suggest that the elevated endogenous levels of riboflavin and hypoxanthine observed in plasmid-carrying strains may directly contribute to their reduced fitness. Given the role of riboflavin-derived cofactors in electron transport chain function and the redox-active nature of hypoxanthine, we propose that the fitness cost imposed by pMDRHI2 carriage in *S*. Indiana and *S*. Enteritidis strains is partially attributable to disruption of aerobic respiratory and redox homeostasis.

In conclusion, this study reveals that serovar-specific host factors drive the fitness costs of IncHI2 plasmid pMDRHI2 in *Salmonella*. Mechanistically, plasmid carriage consistently reprogrammes bacterial microcompartment-associated operons (*pdu*/*eut*), sulphur and glycerophospholipid metabolism genes, leading to convergent disruptions in propanoate metabolism, aerobic respiration and redox balance – yet these effects are distinctly serovar-dependent. A key limitation is the use of a single transconjugant per strain, precluding separation of plasmid effects from conjugation-associated mutations. Future studies generating multiple independent transconjugants are needed to elucidate how plasmid-mediated metabolic reprogramming translates into host-specific fitness costs.

## Supplementary material

10.1099/mgen.0.001715 Supplementary Material 1.

## Abbreviations

ARG: antibiotic resistance gene
ARGs: antibiotic resistance genes
AUC: area under the growth curve
DEGs: differentially expressed genes
DEMs: differentially expressed metabolites
ESBL: extended-spectrum *β*-lactamase
FC: fold change
FPKM: Fragments Per Kilobase of transcript per Million mapped reads
*gfp*: green fluorescent protein
GO: Gene Ontology
LB: Luria-Bertani
MDR: multidrug-resistant
NTS: non-typhoidal *Salmonella*
OD₆₀₀: OD at 600 nm
RF: relative fitness
RT-qPCR: quantitative reverse transcription PCR
TPM: transcripts per million
VIP: variable importance in projection
