# Supplementary material for: Integrative analysis of fitness and metabolic effects of a large multidrug-resistant plasmid in Salmonella across diverse serovars
Source: Microb Genom. 2026 May 18;12(5):001715. doi: 10.1099/mgen.0.001715 (PMC13183382; doi:10.1099/mgen.0.001715)
Supplement: Supplementary Material 1. [file mgen-12-01715-s001.pdf]

Supplementary Materials for

Integrative analysis of fitness and metabolic effects of a large multidrug-resistant plasmid in *Salmonella* across diverse serovars

Yu-Ting Su [1,2#], Zi-Xuan Li[1,2#], Peng-Wei Li[1,2], Meng-Ting Yang[1,2], Ruan-Yang Sun[1,2], Ying Xu[1,2], Dong Wang[1,2], Jian Sun[1,2], Xiao-Ping Liao[1,2], Liang-Xing Fang[1,2*]

[1]Guangdong Laboratory for Lingnan Modern Agriculture, National Risk Assessment Laboratory for Antimicrobial Resistance of Animal Original Bacteria, College of Veterinary Medicine, South China Agricultural University, Guangzhou, Guangdong, P. R. China.

[2]Guangdong Provincial Key Laboratory of Veterinary Pharmaceutics Development and Safety Evaluation, South China Agricultural University, Guangzhou, Guangdong, P. R. China.

[#]These authors contributed equally to this work.

[*]Corresponding author: Liang-Xing Fang, E-mail: fanglx@scau.edu.cn.

Keywords: *Salmonella*, multidrug-resistant plasmid, fitness costs, plasmid-chromosome interaction

Supplemental Tables

Table S1. Details of individual isolate accession numbers and isolate metadata

BioSample	Strain	Description	SRA accession number
SAMN46797869	25FS15	DNA sequencing data	SRR32365428
		RNA sequencing data	SRR32326502
SAMN46797870	25FS15/pMDRHI2	DNA sequencing data	SRR32365427
		RNA sequencing data	SRR32326501
SAMN46797871	45	DNA sequencing data	SRR32365426
		RNA sequencing data	SRR32326500
SAMN46797872	45/pMDRHI2	DNA sequencing data	SRR32365425
		RNA sequencing data	SRR32326499
SAMN46797873	D110	DNA sequencing data	SRR32365424
		RNA sequencing data	SRR32326498
SAMN46797874	D110/pMDRHI2	DNA sequencing data	SRR32365423
		RNA sequencing data	SRR32326497
SAMN46797875	L-S3359	DNA sequencing data	SRR32365422
		RNA sequencing data	SRR32326496
SAMN46797876	L-S3359/pMDRHI2	DNA sequencing data	SRR32365421
		RNA sequencing data	SRR32326495
SAMN46797877	ZJM302	DNA sequencing data	SRR32365420
		RNA sequencing data	SRR32326494
SAMN46797878	ZJM302/pMDRHI2	DNA sequencing data	SRR32365419
		RNA sequencing data	SRR32326493

Table S2. Primers used for RT-qPCR

Gene names		primer sequence（5'-3'）
16s	F	ATTAGATACCCTGGTAGTCCACG
	R	CTTGCGGGACTTAACCCAAC
pduA	F	GGGCTATGAAAAGATTGGCTCC
	R	TGTACGGCTTTCACTTCACCCA
pduD	F	GCAGCGATAAACCCGTCTC
	R	CTTCGTCCTGCTGAGTGCC
pduP	F	GTTTGGCGTTATCGGTTCG
	R	CATCTGCTGGGTCGCTTCG
eutE	F	AGTAACGGCAAACGGATGG
	R	CTGATGCGTCTGATGGAAGG
eutN	F	CGCCAGCCACCACTTCATC
	R	ACGACCCAACGGGCAATCC
eutM	F	ACGTGTACGGAGACCAACTC
	R	CTCCGATGCGATGGTAAAAGC

Table S3. Parameters of RT-qPCR

Gene	Parameters of PCR reaction
16s	（95℃, 30s）+{（95℃, 10s）+（60℃, 30s）+（72℃, 30s）}×40+（95℃,10s）+（65℃,60s）+（97℃,1s）+（37℃,30s）
pduA	（95℃, 30s）+{（95℃, 10s）+（60℃, 30s）+（72℃, 30s）}×40+（95℃,10s）+（65℃,60s）+（97℃,1s）+（37℃,30s）
pduD	（95℃, 30s）+{（95℃, 10s）+（60℃, 30s）+（72℃, 30s）}×40+（95℃,10s）+（65℃,60s）+（97℃,1s）+（37℃,30s）
pduP	（95℃, 30s）+{（95℃, 10s）+（60℃, 30s）+（72℃, 30s）}×40+（95℃,10s）+（65℃,60s）+（97℃,1s）+（37℃,30s）
eutE	（95℃, 30s）+{（95℃, 10s）+（60℃, 30s）+（72℃, 30s）}×40+（95℃,10s）+（65℃,60s）+（97℃,1s）+（37℃,30s）
eutN	（95℃, 30s）+{（95℃, 10s）+（60℃, 30s）+（72℃, 30s）}×40+（95℃,10s）+（65℃,60s）+（97℃,1s）+（37℃,30s）
eutM	（95℃, 30s）+{（95℃, 10s）+（60℃, 30s）+（72℃, 30s）}×40+（95℃,10s）+（65℃,60s）+（97℃,1s）+（37℃,30s）

Table S4. The top 17 most-expressed chromosomal genes in the *Salmonella* in response to plasmid pMDRHI2 carriage

Gene	log_2(TPM)					Gene function
	ZJM302	L-S3359	D110	25FS15	45	
cspC	13.73	15.09	14.40	8.41	13.99	Cold shock-like protein CspC
ibpB	11.62	12.73	14.15	5.51	14.41	Small heat shock protein IbpB
ibpA	12.19	13.34	14.03	3.44	15.05	Small heat shock protein IbpA
grcA	12.32	12.94	13.43	0	14.67	Autonomous glycyl radical cofactor GrcA, stress-induced glycyl radical protein that can replace an oxidatively damaged pyruvate formate-lyase subunit
bhsA	9.76	11.85	12.17	0	14.28	Multiple stress resistance protein BhsA
rpmI	13.32	15.08	13.87	1.58	14.01	Similar to *Escherichia coli* 50S ribosomal subunit protein A
infC	12.65	14.36	13.39	1.59	13.53	IF-3 has several functions that are required and promote translation initiation including preventing association of 70S by binding to 30S
rplT	12.70	14.27	13.13	1.58	13.18	Binds directly to 23S ribosomal RNA prior to *in vitro* assembly of the 50S ribosomal subunit
raiA	12.43	13.06	15.22	0	13.49	Ribosome-associated inhibitor A
ompA	12.77	14.32	13.69	4.86	13.97	Outer membrane protein A
ompD	12.79	14.19	13.16	0	11.33	Outer membrane porin protein OmpD
lpp1	13.81	13.96	14.05	6.33	13.94	Major outer membrane lipoprotein Lpp 1
ibpB	11.62	12.73	14.15	5.51	14.41	Major outer membrane lipoprotein Lpp
*ahpC*_1	10.38	11.28	12.85	15.49	11.65	Alkyl hydroperoxide reductase C
group_ 2731	14.64	15.57	15.43	0	0	hypothetical protein
group_ 3513	0	0	14.75	0	0	hypothetical protein
group_ 3514	0	0	14.99	0	0	hypothetical protein

group_ 7995	0	0	0	15.49	0	hypothetical protein
group_ 9690	0	0	0	0	16.34	hypothetical protein

“0” means the corresponding gene was not present in the strains.

Table S5. The top 12 most-expressed plasmid genes in the *Salmonella* harboring pMDRHI2

Gene	Strain					Gene function
	ZJM302	L-S3359	D110	25FS15	45	
nimC	+	+	+	+	+	NimC/NimA family protein
aac(3)-IV	+	+	+	+	+	Aminoglycoside N(3)-acetyltransferase
orf82	+	+	+	+	+	hypothetical protein
bla$_{CTX\text{-}M\text{-}14}$	+	+	+	+	+	extended-spectrum beta-lactamase; CTX-M family
aph(4)-Ia	+	+	+	+	+	Aminoglycoside 4-phosphotransferase; APH(4)-I
dfrA12	+	+	+	+	+	Dihydrofolate reductase
sul2	+	+	-	-	+	Dihydropteroate synthase type-2
estX	+	+	+	+	+	Streptothricin acetyltransferase
h-ns	+	+	+	+	-	DNA-binding protein H-NS
mcr-1	+	+	-	+	+	Lipid A phosphoethanolamine transferase; potentially related to polymyxin resistance protein MCR-1
IS*6*	+	+	+	-	+	Mobile element protein
repHI2	+	-	+	-	+	Replication protein A

"+" and "-" means that transcript abundance in $\log_2$ of transcripts per million (TPM) of plasmid genes > 14 and < 14, respectively.

Table S6. Sequence comparison analysis of the *pdu* and *eut* operons from 5 tested *Salmonella* strains at nucleotide level

Strain	Serovar	*pduFABCDEGHJKLMNOPQSTUVWX* (18.088 kb)		*eutSPQTDMNEJGHABCLKR* (15.267 kb)	
		identity %	coverage%	identity %	coverage %
ZJM302	1,4,[5],12:i:-	99.97	99.99	99.98	100
L-S3359	1,4,[5],12:i:-	99.98	99.99	99.98	100
D110	Typhimurium	99.98	99.99	99.97	100
25FS15	Indiana	97.87	99.99	98.81	91.6
45	Enteritidis	98.28	99.99	98.77	91.87

Annotation: The nucleotide sequence of the *pdu* and *eut* operons from the standard *Salmonella* Typhimurium ATCC 14028 bacteria was used as the reference sequence.

Table S7. Sequence comparison analysis of the genes related to the endogenous synthesis pathway for Cys from 5 tested *Salmonella* strains at nucleotide level

Gene	Strain	ZJM302	L-S3359	D110	25FS15	45
	Serovar	1,4,[5],12:i:-	1,4,[5],12:i:-	Typhimurium	Indiana	Enteritidis
cysAWUP	Identity (%)	99.97	99.97	100	98.4	98.69
	Coverage (%)	100	100	100	99.92	100
cysCND	Identity (%)	99.97	99.97	100	98.58	99.22
	Coverage (%)	100	100	100	100	100
cysHIJ	Identity (%)	100	100	100	93.59	95.44
	Coverage (%)	100	100	100	99.93	99.95
cysE	Identity (%)	100	100	100	99.64	99.76
	Coverage (%)	100	100	100	100	100
cysK	Identity (%)	100	100	100	99.28	99.38
	Coverage (%)	100	100	100	100	100

Annotation: The nucleotide sequence of the genes related to the endogenous synthesis pathway for Cys from the standard *Salmonella* Typhimurium ATCC 14028 bacteria was used as the reference sequence.

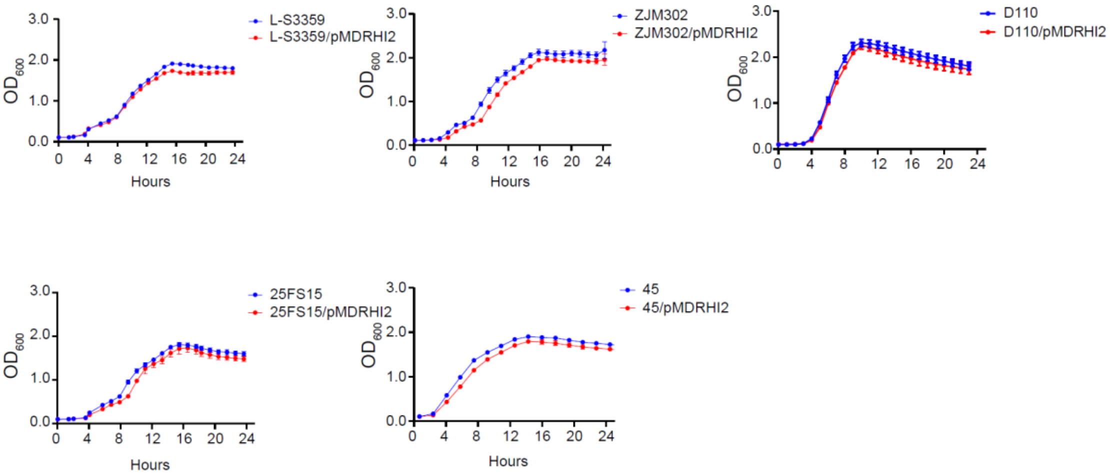

Figure S1. Growth curves for plasmid-carrying transconjugants and the corresponding plasmid-free parent recipients

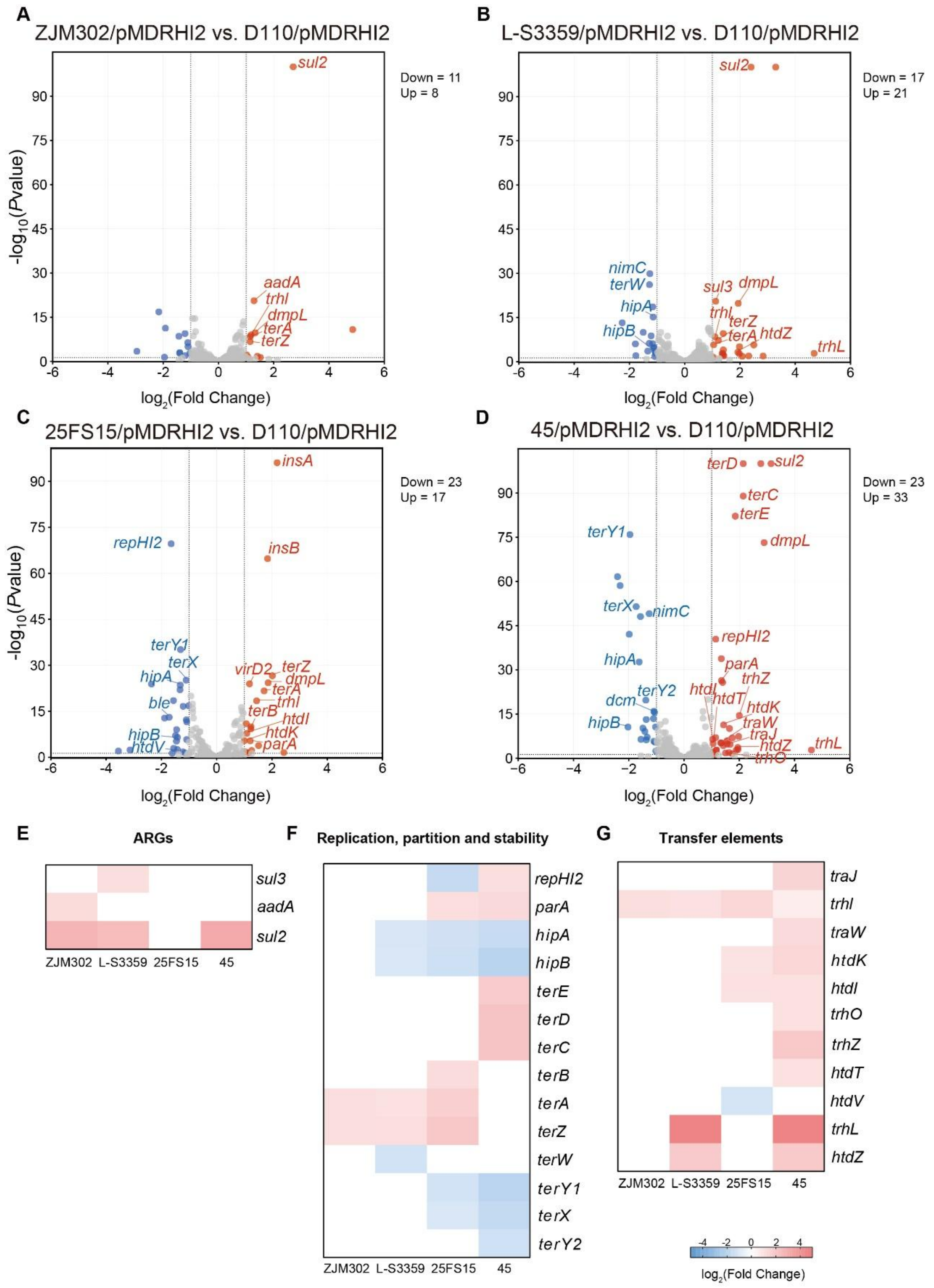

Figure S2. Plasmid-derived gene expression in *Salmonella* hosts of differing serovars

(A-D) Transcriptome profiles for plasmid pMDRHI2 in **(A)** 1,4,[5],12:i:- ZJM302 and **(B)** L-S3359 strains, **(C)** Indiana 25FS15 strain and **(D)** Enteritidis45 strain relative to

the reference Typhimurium D110 strain;

(E-G) Gene expression analysis of pMDRHI2 plasmid-encoded **(E)** ARGs, **(F)** replication, partition and stability genes and **(G)** transfer elements.

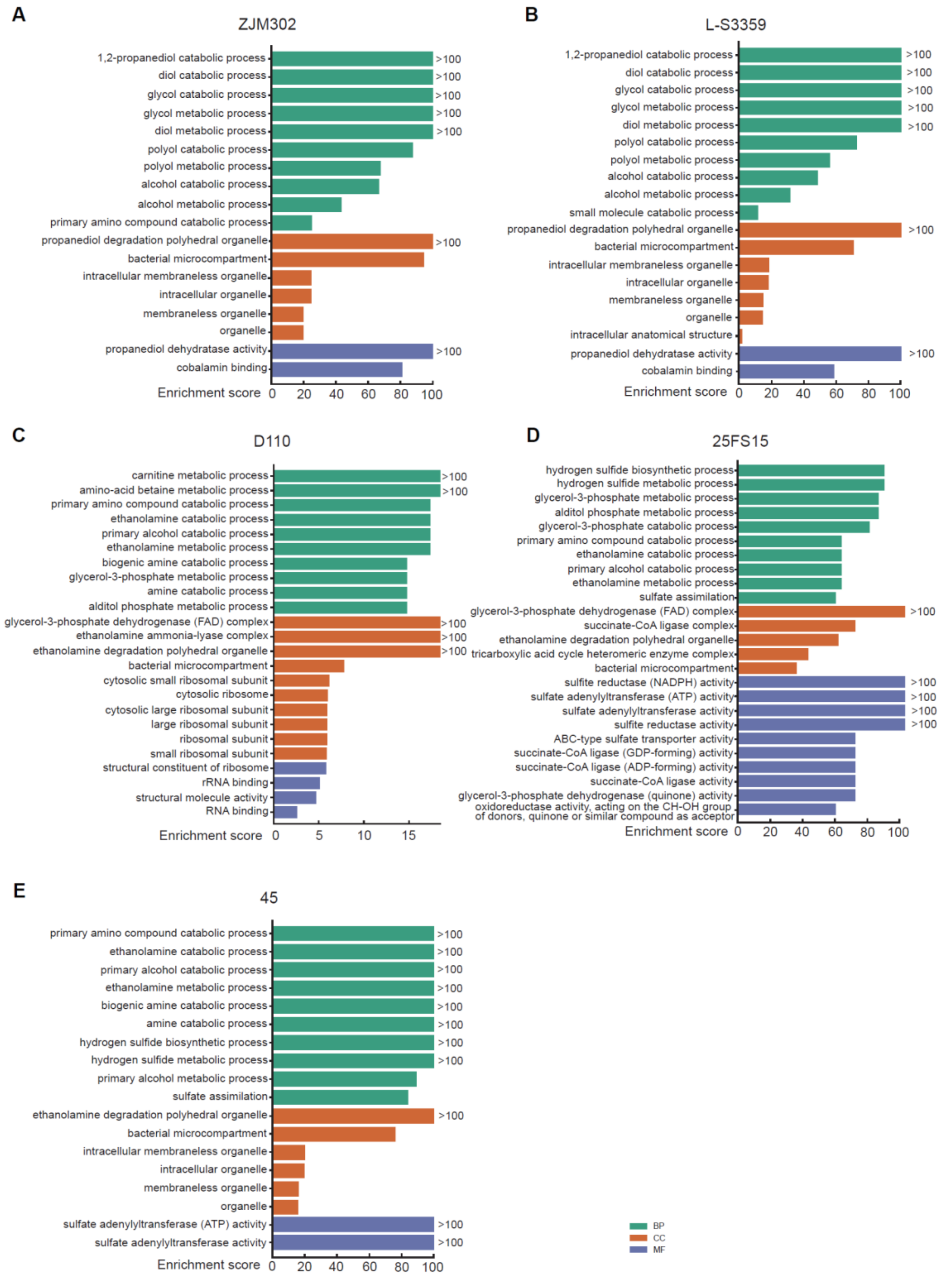

Figure S3. Gene Ontology (GO) annotation enrichment analysis using all differentially-expressed genes (DEGs) from each of five pMDRHI2 plasmid-carrying *Salmonella* strains relative to that of the corresponding plasmid-free *Salmonella* strains

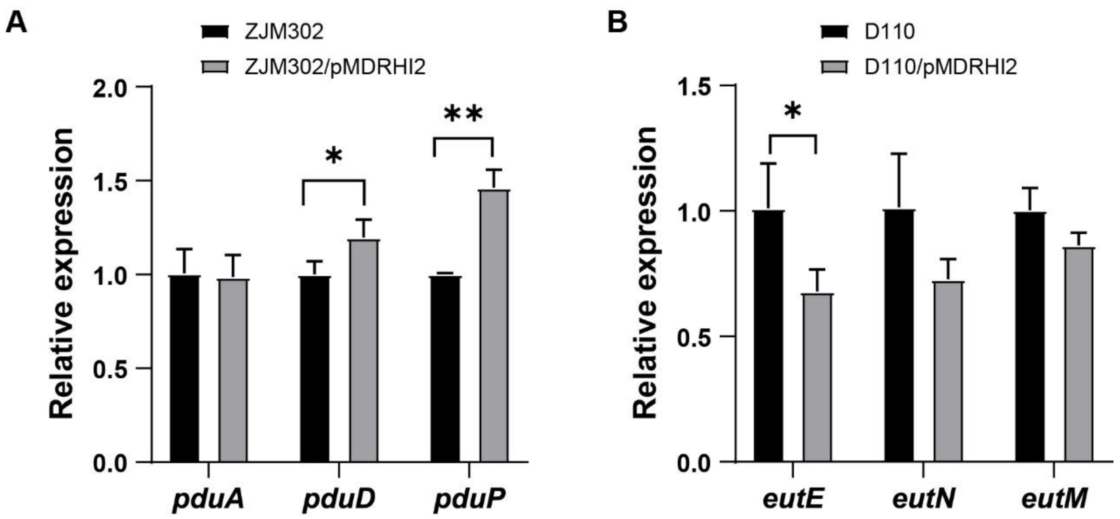

Figure S4. The relative mRNA expression levels of *pdu (A)* and *eut (B)* genes using RT-qPCR

"*" represents "$p < 0.05$", "**" represents "$p < 0.01$".

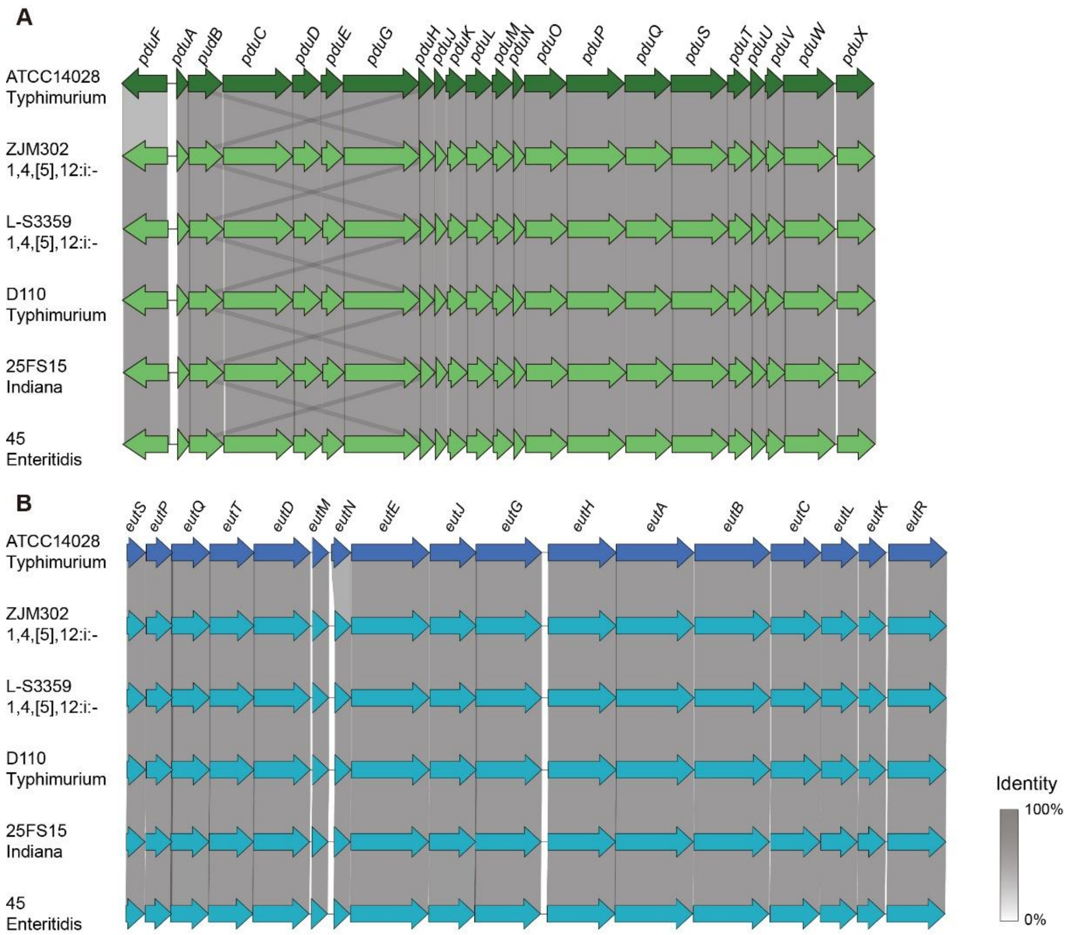

Figure S5. Sequence comparison analysis of the *pdu* (A) and *eut* (B) operons from 5 tested *Salmonella* strains at nucleotide level

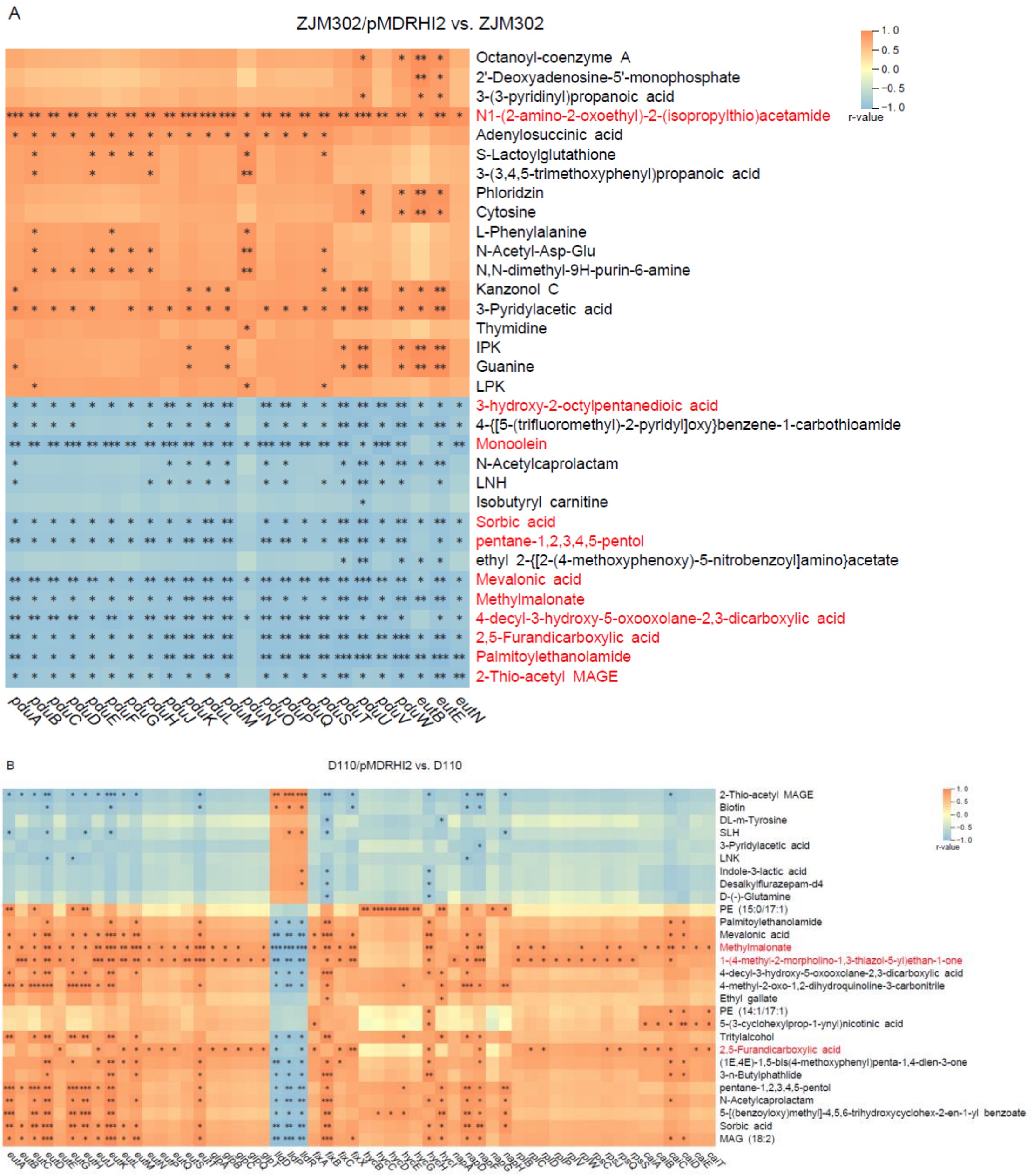
A
ZJM302/pMDRHI2 vs. ZJM302
Octanoyl-coenzyme A
2'-Deoxyadenosine-5'-monophosphate
3-(3-pyridinyl)propanoic acid
N1-(2-amino-2-oxoethyl)-2-(isopropylthio)acetamide
Adenylosuccinic acid
S-Lactoylglutathione
3-(3,4,5-trimethoxyphenyl)propanoic acid
Phloridzin
Cytosine
L-Phenylalanine
N-Acetyl-Asp-Glu
N,N-dimethyl-9H-purin-6-amine
Kanzonol C
3-Pyridylacetic acid
Thymidine
IPK
Guanine
LPK
3-hydroxy-2-octylpentanedioic acid
4-{[5-(trifluoromethyl)-2-pyridyl]oxy}benzene-1-carbothioamide
Monoolein
N-Acetylcaprolactam
LNH
Isobutyryl carnitine
Sorbic acid
pentane-1,2,3,4,5-pentol
ethyl 2-{[2-(4-methoxyphenoxy)-5-nitrobenzoyl]amino}acetate
Mevalonic acid
Methylmalonate
4-decyl-3-hydroxy-5-oxooxolane-2,3-dicarboxylic acid
2,5-Furandicarboxylic acid
Palmitoylethanolamide
2-Thio-acetyl MAGE
pduA
pduB
pduC
pduD
pduE
pduF
pduG
pduH
pduJ
pduK
pduL
pduM
pduN
pduO
pduP
pduQ
pduS
pduT
pduU
pduV
pduW
eutB
eutE
eutN
1.0
0.5
0.0
-0.5
-1.0
r-value
B
D110/pMDRHI2 vs. D110
2-Thio-acetyl MAGE
Biotin
DL-m-Tyrosine
SLH
3-Pyridylacetic acid
LNK
Indole-3-lactic acid
Desalkylflurazepam-d4
D-(-)-Glutamine
PE (15:0/17:1)
Palmitoylethanolamide
Mevalonic acid
Methylmalonate
1-(4-methyl-2-morpholino-1,3-thiazol-5-yl)ethan-1-one
4-decyl-3-hydroxy-5-oxooxolane-2,3-dicarboxylic acid
4-methyl-2-oxo-1,2-dihydroquinoline-3-carbonitrile
Ethyl gallate
PE (14:1/17:1)
5-(3-cyclohexylprop-1-ynyl)nicotinic acid
Tritylalcohol
2,5-Furandicarboxylic acid
(1E,4E)-1,5-bis(4-methoxyphenyl)penta-1,4-dien-3-one
3-n-Butylphathlide
pentane-1,2,3,4,5-pentol
N-Acetylcaprolactam
5-[(benzoyloxy)methyl]-4,5,6-trihydroxycyclohex-2-en-1-yl benzoate
Sorbic acid
MAG (18:2)
1.0
0.5
0.0
-0.5
-1.0
r-value

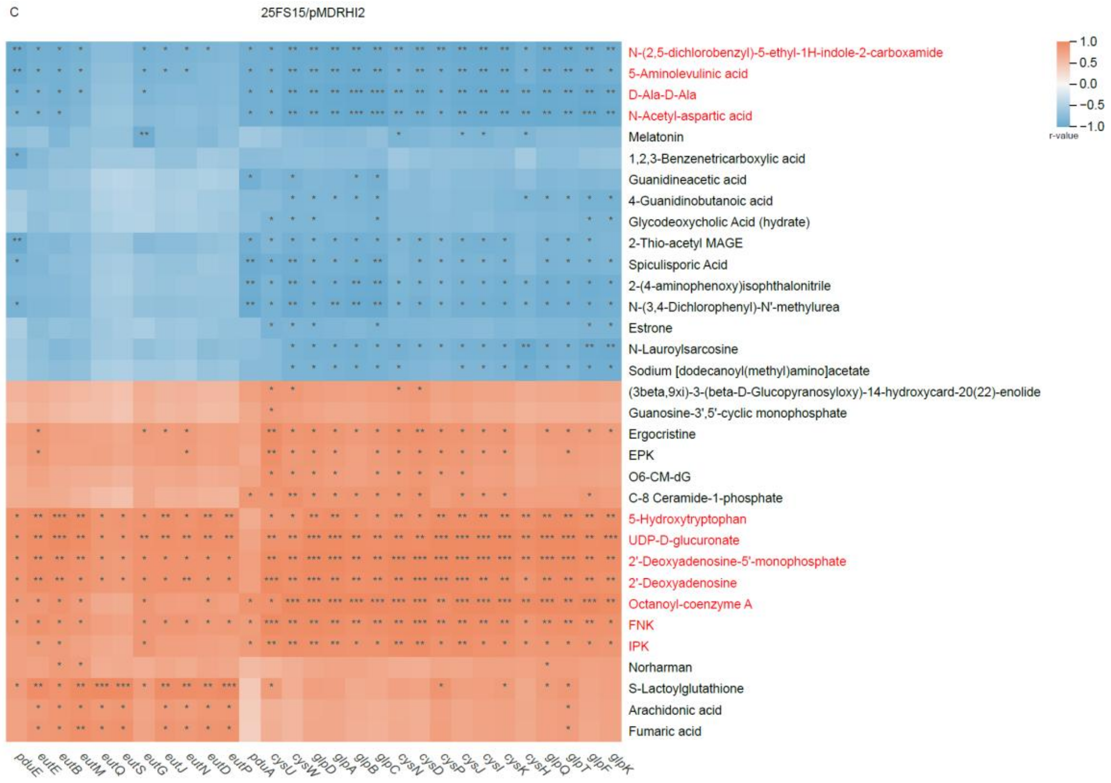

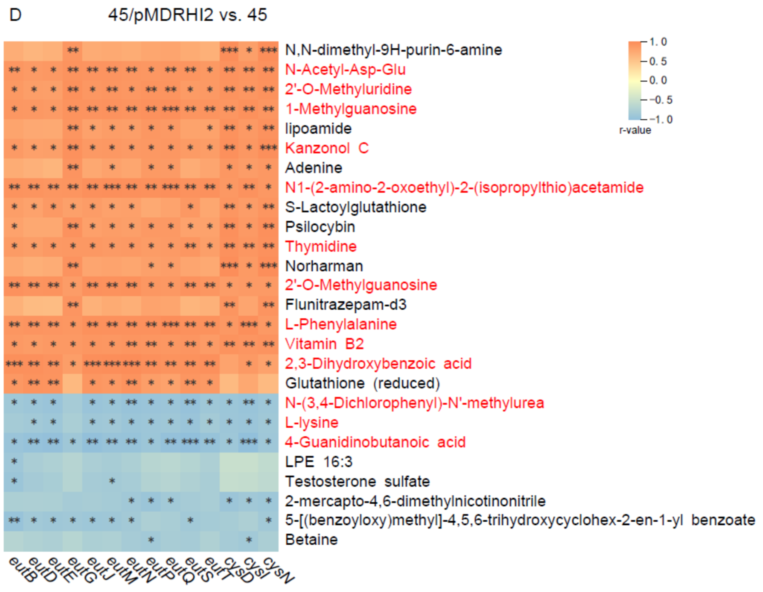

Figure S6. Systematic molecular correlations across multi-omics integrating carriage of plasmid mediated changes in metabolome and transcriptome

Interaction network (Spearman coefficient ≥ ± 0.8) showing significant correlations between the top 40 differentially-expressed metabolites (DEMs) and the major differentially-expressed genes (DEGs) in each of 4 pMDRHI2 plasmid-carrying *Salmonella* strains relative to that of corresponding plasmid-free strains. The DEMs

marked in red represent the DEMs that was significantly positively or negatively correlated with most of the major differentially-expressed genes (DEGs) in each case. "*" represents "$p < 0.05$", "**" represents "$p < 0.01$", "***" represents "$p < 0.001$".

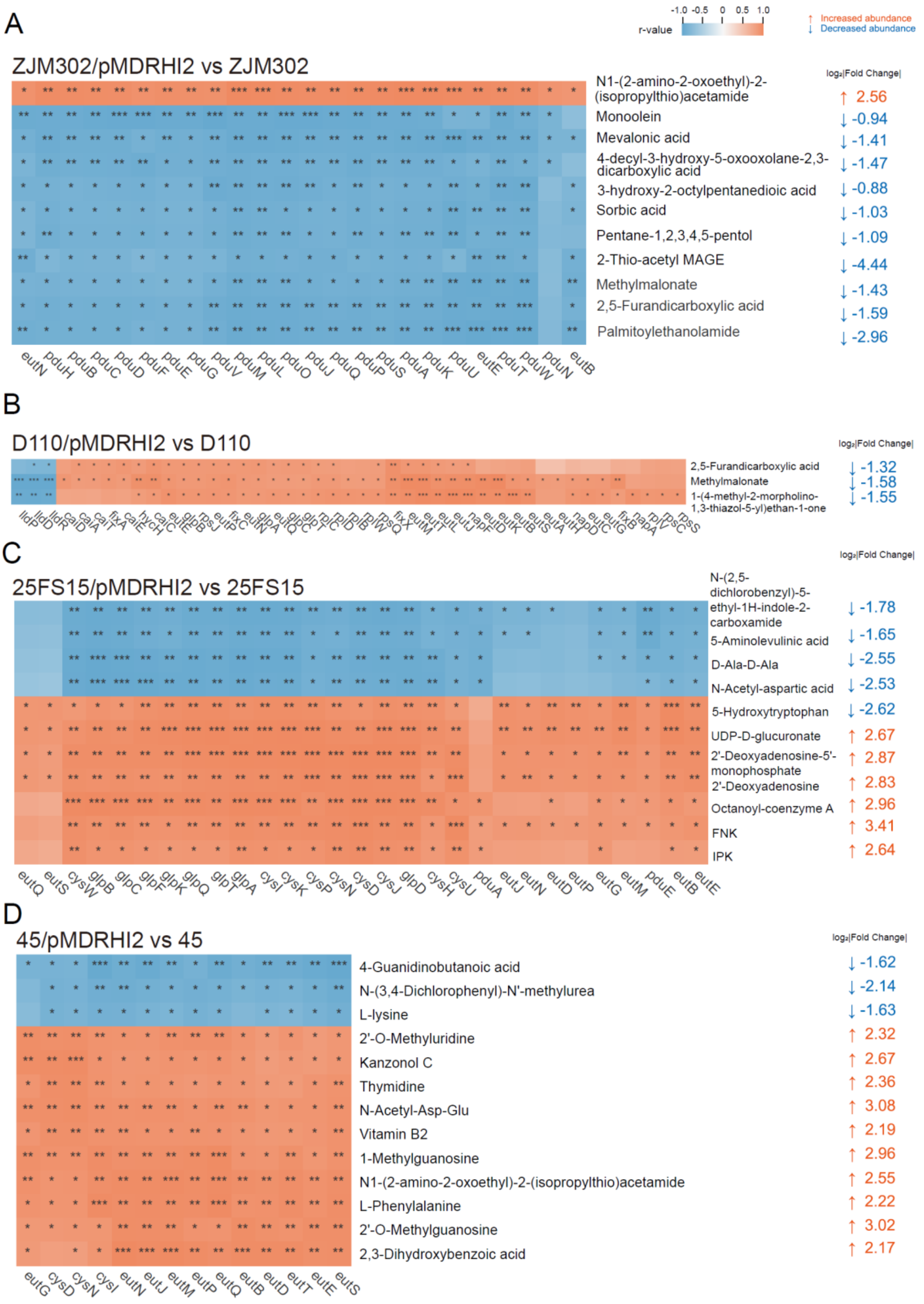

Figure S7. Part of the top 40 differentially-expressed metabolites (DEMs) was significantly positively or negatively correlated with most of the major differentially-expressed genes (DEGs) in each of 4 pMDRHI2 plasmid-carrying *Salmonella* strains relative to that of corresponding plasmid-free strains

“*” represents “$P < 0.05$”, “**” represents “$P < 0.01$”, “***” represents “$P < 0.001$”. The values ($\log_2$ (Fold change)) represent the abundances of DEMs.
